# Overdose response centering inequity and diversity study: a protocol for assessing the population-level and equity impact of the emergency medical services system changes using critical race theory

**DOI:** 10.3389/fpubh.2025.1629518

**Published:** 2025-09-15

**Authors:** Ohshue S. Gatanaga, Nicholas Cotta, Kimiam Waters, Alden Gu, Omeid Heidari, Tessa Frohe, Courteney Wettemann, Andre Morris, Esther Rourke, India Ornelas, Malika Lamont, Deaunte Damper, Callan Fockele, Emily C. Williams, Jenna van Draanen

**Affiliations:** ^1^Department of Health Systems and Population Health, University of Washington School of Public Health, Seattle, WA, United States; ^2^College of Arts and Sciences, University of Washington, Seattle, WA, United States; ^3^Department of Child, Family, and Population Health Nursing, University of Washington School of Nursing, Seattle, WA, United States; ^4^Department of Psychiatry and Behavioral Sciences, University of Washington School of Medicine, Seattle, WA, United States; ^5^Voices of Color Advocating for Liberty – Washington, Seattle, WA, United States; ^6^Department of Emergency Medicine, University of Washington School of Medicine, Seattle, WA, United States; ^7^Downtown Emergency Services Center, Seattle, WA, United States

**Keywords:** structural racism, overdose, emergency medical services, racial disparities, opioid mortality, community-based participatory research

## Abstract

**Background:**

Structural racism continues to drive racial disparities in opioid-related deaths by creating inequitable access to healthcare, shaping prescription practices, limiting availability of culturally responsive care, and concentrating socioeconomic disadvantage in racial/ethnic minority communities. Emergency Medical Services (EMS) based interventions provide a critical opportunity to address these disparities at the frontlines of care, as minoritized communities often utilize EMS as their usual source of care. In King County, Washington, EMS has begun implementing several system changes aimed at reducing opioid overdose deaths, promoting harm reduction strategies, increasing access to overdose prevention resources, and improving outcomes for individuals who survive overdoses. The Overdose Response Centering Inequity and Diversity (ORCID) study will evaluate these EMS system changes to understand their impact on opioid-related outcomes differentially by race and ethnicity.

**Methods:**

This study employs a mixed-methods, hybrid effectiveness-implementation design with three aims: (1) to understand experiences and outcomes for minoritized racial groups at the patient level using a prospective cohort study (*n* = 500) of non-fatal overdose survivors; (2) to evaluate EMS system changes’ implementation processes from the perspectives of Black, Hispanic/Latinx, and American Indian/Alaska Native non-fatal overdose survivors using in-depth interviews (*n* = 60); and (3) to examine population-level impacts of EMS system changes on racial disparities using secondary data from King County EMS. Utilizing an innovative community-based participatory approach, this study centers and incorporates individuals with lived and living experience of drug use as equal partners throughout the research process.

**Discussion:**

Through a rigorous evaluation of EMS system changes in King County, this study will generate actionable insights for improving EMS responses to the opioid epidemic and addressing racial disparities both locally and nationally. As one of the first studies to track a longitudinal cohort of non-fatal overdose survivors, ORCID will provide critical data on both short- and long-term outcomes, informing future interventions focused on improving continuum of care for overdose survivors. By employing a community-engaged approach, the study centers the lived experiences of those most affected and enhances the relevance of the study findings. Potential limitations include the rapidly evolving landscape of EMS interventions and biases associated with non-random sampling.

## Introduction

In the United States (U. S.), the public health crisis of overdose continues unabated with racial disparities in opioid-related outcomes. Specific to King County, Washington, overdose deaths increased 32.7% from 1,009 deaths in 2022 to 1,359 deaths in 2023 (1). Among minoritized racial/ethnic individuals, defined as those who do not identify as exclusively White, 2023 opioid-related death rates were 281.2 (per 100,000) for American Indian/Alaskan Native (AI/AN) people, 165.2 for Black people, and 60.9 for Hispanic people, compared to 50.7 for White people (1). Structural racism—defined as the “totality of ways in which societies foster racial discrimination through mutually reinforcing systems of housing, education, employment, earnings, benefits, credit, media, health care, and criminal justice”—contributes to the disproportionate impacts of the opioid epidemic on racial/ethnic minority communities (2–6). Policies, including a nationwide response focusing on opioid overdose primarily among White individuals and the targeted roll-out of buprenorphine to these communities thereafter (7, 8), are thought to have widened the well-documented racial disparities in essential treatment and harm reduction services (9, 10).

Structural changes, such as policies and other community practices that allocate resources to communities most impacted by opioid-related disparities, are necessary to address racial equity. Emergency Medical Service (EMS) settings may offer opportunities for providing such solutions, as EMS providers are frontline responders to opioid overdose, and racial/ethnic minority communities more frequently report utilizing EMS as their source of healthcare compared to White individuals (11). In King County, approximately 40% of individuals who died of overdose in 2018 had at least one prior EMS encounter in the previous year (12). To this extent, one of the greatest predictors of repeat overdose and death in the following year is a prior non-fatal overdose (13, 14). As such, EMS-delivered interventions may be an important tool to alleviate disparities in access to evidence-based practices (EBPs) among racial/ethnic minoritized people who use drugs (PWUD) (9, 10).

Practical implementation of EBPs within the EMS setting necessitates multilevel interventions, as racial discrimination occurs at the interpersonal, community, and structural levels (15, 16). At the structural level, EBPs for preventing overdose deaths and improving substance use treatment outcomes, including leave-behind naloxone, fentanyl test strips, and buprenorphine treatment, remain limited in their implementation in community settings and contribute to inequitable access for racial/ethnic minority communities (9, 10, 17–19). At the interpersonal level, structural racism manifests in EMS settings through stigma and discrimination toward PWUD and racialized groups during encounters. First responders may be reluctant to engage in overdose prevention services (10), and PWUD have cited discriminatory experiences with first responders that are further pronounced among minoritized racial/ethnic PWUD (20–22). Thus, interventions that simultaneously address first responder stigma toward racialized PWUD, as well as system-level changes that introduce EBPs and increase access to life-saving resources, may address several sequelae of structural racism in the opioid epidemic (7).

Alongside implementation of multilevel EBPs, interventions addressing overdose-related outcomes benefit greatly from involving PWUD in the design, implementation, and evaluation of EBPs. Historically, PWUD and other marginalized communities have been excluded from engaging in evaluation and research processes, leading to research with reduced relevance to the needs of PWUD and diminished trust toward academic and public institutions (23). By ensuring that research goals and processes reflect the lived experiences of PWUD, community-based participatory research (CBPR) has demonstrated significant improvements in health programs, policy, and health equity as it relates to substance use outcomes (24–27).

Recognizing the potential for both EMS and CBPR-based interventions, Public Health Seattle and King County (PHSKC) developed an opioid overdose response program leveraging the expertise of the Research with Expert Advisors on Drug Use (READU) team, a diverse community-engaged partnership of academic researchers, PWUD community members, EMS leaders, public health officials, and clinicians. The resulting intervention incorporated system changes aimed at reducing opioid overdose deaths, promoting harm reduction strategies, increasing access to overdose prevention resources, and improving outcomes for individuals who survive overdoses in King County, WA. The intervention components include training for all EMS providers on stigma reduction and trauma-informed care, an EMS naloxone leave-behind program with fentanyl strips, and a warm hand-off to follow-up teams for connecting overdose survivors to substance use treatment. Adopting these programs iteratively, King County EMS plans to have 90% of its teams participating in this intervention by 2026. Alongside the PHSKC intervention, several other significant system changes are occurring with the potential to impact disparities in overdose response: (1) the option of EMS-initiated buprenorphine treatment (for opioid use disorder) in King County; (2) the creation of an overdose-specific mobile integrated health team to respond to overdose events in the Seattle downtown area; (3) the development of an-overdose specific receiving center in Seattle as an alternative to the emergency department; and (4) the launch of five crisis solution centers serving as additional sites for linkage and care post-overdose focusing on behavioral health crises.

The current study, Overdose Response Centering Inequity and Diversity (ORCID) study, will evaluate several EMS system changes to inform efforts to combat the opioid epidemic and address disparities arising from structural racism. Capitalizing on the natural experimental conditions provided by EMS system changes, ORCID will examine the impact of EMS system changes on health outcomes in a hybrid implementation-effectiveness study. Through partnerships with community researchers who have lived and living experiences of drug use, PHSKC, and local EMS providers, this study will expand current understanding of the lived experiences of racial/ethnic minority individuals receiving EMS care and lead to the development and piloting of public health data linkage systems that enable long-term equity surveillance.

## Methods

### Study design and setting

ORCID employs a Type 2 hybrid effectiveness-implementation design to assess the impact of EMS system changes in King County, Washington (28). ORCID includes three aims designed to: (1) understand experiences and outcomes for minoritized racial groups at the patient level using a prospective cohort study of non-fatal overdose survivors; (2) evaluate EMS system changes’ implementation processes from the perspectives of Black, Hispanic/Latinx, and American Indian/Alaska Native non-fatal overdose survivors using in-depth interviews; and (3) examine population-level impacts of EMS system changes on racial disparities using secondary data from King County EMS. This concurrent triangulation design leverages the strengths of both quantitative and qualitative methods to understand implementation processes and test system change effectiveness (29):

Aim 1 utilizes a prospective cohort of non-fatal overdose survivors (recruitment goal: *n* = 500), with linked data across multiple institutions and systems, followed at three time points (baseline, 6 months post-, and 12 months post-baseline) to provide understanding of experiences and outcomes for minoritized racial/ethnic groups at the patient level. Aim 1 involves primary data collection from individuals with overdoses in the past 6 months responded to by King County EMS.
Aim 2 further evaluates the EMS system changes’ implementation processes through in-depth interviews with a subset of the prospective cohort who are Black, Hispanic/Latinx, and/or AI/AN non-fatal overdose survivors (recruitment goal: *n* = 60). These interviews will be designed to understand perceived stigma, quality of overdose reversal care, and perceived value of the PHSKC intervention components (i.e., leave-behind naloxone, fentanyl strips, and experiences of care linkage) and other EMS system changes at the patient level for these minoritized racial/ethnic groups.
Aim 3 uses accessible data from King County EMS to conduct secondary data analysis on the impact of EMS system changes on racial disparities in population-level outcomes via an interrupted time series design with switched replication (30, 31).

### Conceptual framework

Through engagement with community partners, the study team proposes a conceptual framework for ORCID rooted in the Consolidated Framework for Implementation Research (CFIR) and Public Health Critical Race Praxis (PHCRP) (32, 33).

**Figure 1:**
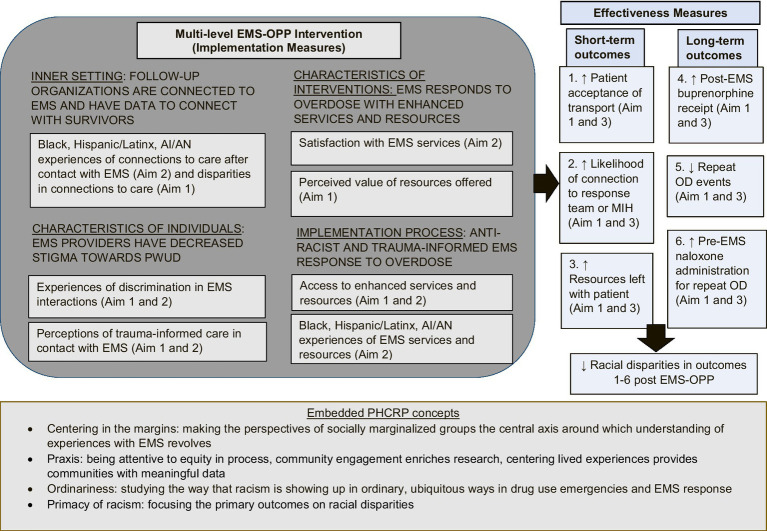
Conceptual framework rooted in CFIR and critical race theory and study measures.

CFIR supports the assessment of the multilevel, EMS system changes’ inner setting, characteristics of individuals, intervention characteristics, and implementation processes, and its expected outcomes. In addition, PHCRP facilitates the study team’s conceptualization of structural racism and discrimination as root causes of social inequities. PHCRP provides guiding principles for antiracist work including: (1) understanding race as a social construct and racialization as a fundamental contributor to disparities; (2) employing race consciousness as opposed to “colorblindness”; (3) ensuring discourse surrounding racial inequities should prioritize the experiences and perspectives of people from marginalized groups; (4) considering how macro-level social forces impact health outcomes and how multiple identities interact to impact health; and (5) critiquing knowledge production and disciplinary conventions, including researchers’ biases. Specific to ORCID, essential PHCRP concepts that are embedded include ensuring the experiences with EMS are centered around the needs of racially and socially marginalized groups, being attentive to equitable participation in research processes, studying how racism shows up in ubiquitous ways in substance use-related emergencies and EMS responses, and focusing study outcomes on racial disparities and equity (Figure 1).

Consistent with our conceptual framework, the study measures include several domains of CFIR. Specifically, the Aim 1 study includes implementation measures of the inner setting (i.e., experiences of connections of care), characteristics of individuals impacted by overdose (i.e., discrimination in EMS interactions and perception of EMS trauma-informed care), the characteristics of EMS-based interventions (i.e., perceived value of EMS-based resources offered), and the implementation process (i.e., access to enhanced EMS services and resources). Additionally, we include short-term and long-term effectiveness outcomes measuring the acceptance of EMS transportation, connection to follow-up teams, offered resources, post-EMS buprenorphine receipt, pre-EMS naloxone administration, and repeated overdose. The Aim 2 interviews elicit additional implementation measures including perceptions of EMS trauma-informed care, satisfaction with EMS services, and experiences accessing services and resources. Similar to Aim 1, the secondary data analyses embedded in Aim 3 include effectiveness measures of the short-term and long-term outcomes of EMS system changes. Table 1 includes a visualization of the study measures organized according to the CFIR domains and data sources.

**Table 1:** ORCID study measures organized according to guiding conceptual framework domains and data source.

	Data source		
Construct	Aim 1 prospective cohort	Aim 2 qualitative interviews	Aim 3 king County EMS secondary data
Implementation measures			
Inner setting			
Experience of connections to care	X	X	
Characteristics of individuals			
Discrimination in EMS interactions	X	X	
Perceptions of EMS trauma-informed care	X	X	
Characteristics of interventions			
Perceived value of resources offered	X		
Satisfaction with EMS services		X	
Implementation process			
Access to enhanced services and resources	X	X	
Experiences of accessing services and resources		X	
Effectiveness measures			
Short-term outcomes			
Acceptance of transport	X		X
Connections to follow-up team	X		X
Resources offered:			
Leave-behind naloxone (0/1)	X		X
Fentanyl test strips (0/1)	X		X
Warm hand-off (0/1)	X		X
Long-term outcomes			
Post-EMS buprenorphine receipt	X		X
Pre-EMS naloxone administration	X		X
Repeated overdose	X		X

### Community engagement and development of the current study

ORCID utilizes an innovative CBPR approach to inform every step of the research process (24). Prior to the beginning of study activities, the study team established itself and continues to build upon a unique partnership model, Research with Expert Advisers on Drug Use (READU), where PWUD collaborate as equal partners with academic researchers to develop and execute research around agreed upon core principles (e.g., equitable collaboration, knowledge and action integration, cyclic and iterative processes, co-learning and empowerment, attending to inequities, and building on resources within the community). These principles are intentionally co-created by both PWUD and academic researchers to ensure that the needs and perspectives of PWUD were centered in all aspects of research, thus underscoring the value of equitable participation in shaping substance use policies and programs (34, 35). This approach aligns with PHCRP in “centering the margins” by ensuring that the perspectives of people from marginalized groups are prioritized in the discourse surrounding inequities in substance use-related outcomes.

In ORCID, PWUD community members on READU hold equal decision-making authority with academic researchers. This includes authority in deciding research questions, design, and dissemination of all research projects including ORCID. This shared governance structure is grounded in the recognition that unexamined power dynamics lead to the perpetuation of distrust, disappointment, and exclusion among PWUD and other historically marginalized communities (36). Furthermore, resource allocation is approached with transparency and developed in tandem with community researchers. Community researchers are paid either weekly in cash or/or check depending on their needs and were compensated for a wide range of contributions that are often undervalued or unpaid in traditional academic research settings—including community outreach, relationship-building, and bidirectional trainings.

Leading up to the conception of the study, the study team and READU conducted listening sessions to learn about community experiences with first responders and EMS during overdose encounters, and how structural racism, interpersonal discrimination, and stigma impacted overdose encounters for PWUD. Grounded in PHCRP, which highlights the ubiquity of racism and encourages a structural rather than individual perspective, we posited structural racism and racialization as a fundamental contributor to overdose-related disparities and considered how system-level EMS changes (i.e., macro-level social forces) impacted health outcomes for racial/ethnic minoritized PWUD. In responding to urgent requests from community partners to prioritize racial equity in the implementation and evaluation of substance use-related interventions, the study team partnered with READU, PHSKC, and King County EMS leadership to jointly develop this study proposal. Emergent themes such as variable experiences with first responders, the negative impacts of police response, lack of autonomy during EMS encounters, and increased willingness to engage with people with shared identities informed the study design and development of data collection tools.

The study team subsequently asked the study partners how they would like to participate in guiding the design, implementation, and dissemination of results. The resulting advisory structure was then designed as a community-advisory board (CAB) with two arms: an advisory arm with PWUD who identify with the minoritized racial groups included in ORCID, harm reduction and community service organizations, EMS partners and PHSKC representatives; and an action arm with up to five peer researchers with lived and living experience of drug use. For the CAB advisory arm, members give advice, guidance, and offer consultation on research plans; assist in securing data collection sites; aid in recruitment; and support widespread dissemination activities. For the action arm, CAB members recruited from READU, who are subsequently trained in research methods, take an active role in conducting recruitment, screening, data collection, analysis, interpretation, and presentation of study findings.

### Peer support research coordinators

In addition to the advisory and action arms of the CAB, the ORCID study team includes Peer Support Research Coordinators (PSRC), several of whom are Certified Peer Counselors (CPC) accredited by the Washington State Health Care Authority. Peer support is an evidence-based approach that leads to improved health outcomes among PWUD, and especially for racially marginalized PWUD through broadened cultural relevance (37, 38). As such, PSRC bring skills around motivational interviewing, trauma-informed care, effective storytelling, crisis care, and goal setting to create a safe and nonjudgmental space that reduces the risk of traumatization for participants via survey questions and qualitative interviews.

All PSRC participate in a structured onboarding process, which includes multi-day training focused on the study protocol, research ethics, harm reduction principles, trauma-informed engagement, and culturally responsive peer support. To ensure consistency and fidelity across study sites, PSRC attend weekly READU meetings, which include scheduled training sessions on standardized data collection procedures led by lead study staff, as well as instruction on Peer Support Specialist (PSS) concepts and tools facilitated by licensed psychologists. PSRC facilitate bi-directional training to refine and demonstrate their understanding of PSS concepts through collaborative learning and shared reflection. Additionally, PSRC are provided with ongoing opportunities for one-on-one learning sessions with licensed clinicians designed to provide emotional and clinical support. PSRC also receive regular supervision from study staff with expertise in both research methods and peer-led models of care. Supervision includes debriefing sessions to reflect on participant interactions and troubleshoot existing challenges with peer support provision.

In practice, PSRC support study objectives through various means: they provide post-interview and post-survey care as needed, minimizing re-traumatization that participants may experience from data collection. This process includes asking reflective, supportive questions post-interview on an as-needed basis and providing appropriately tailored resources and ongoing support for participants expressing concerns outside of data collection. In instances where participants become activated by questionnaire content, PSRC validate the emotional heaviness of the questions and leverage participants’ coping strategies and strengths via motivational interviewing (39). Finally, peer support is integral to data collection and recruitment site identification given PSRCs’ lived experiences, community knowledge, and familiarity with underrepresented populations and environments. For example, PSRC guide efforts to identify and reach individuals within encampments, community gathering spaces, or service provider locations, ensuring inclusive representation of PWUD who face multiple barriers to research participation in the study.

### Aim 1: study sample and recruitment

For Aim 1, the study team will collect primary data by recruiting up to 500 individuals to join a prospective cohort of overdose survivors. Eligible individuals will be those who: (1) are at least 18 years old; (2) are English-speaking; (3) have experienced a recent (with the past 6 months) non-fatal overdose in King County, to which EMS responded. The study team will conduct purposive sampling to oversample participants with minoritized racial/ethnic identities most impacted by overdoses in King County (i.e., Black, Hispanic/Latinx, AI/AN overdose survivors). We will follow up with participants at two time points (i.e., 6- and 12-months post-initial contact) to develop an understanding of experiences and outcomes for minoritized racial/ethnic groups at the individual level.

Recruitment of overdose survivors will be done in partnership with King County EMS, as well as mobile integrated health response units (e.g., Health One/Health 99) and paramedic programs (i.e., Medic One) embedded within the Seattle Fire Department. The study team will also recruit via other channels available to them, including but not limited to outreach via a large university-based medical center, PHSKC’s post-overdose response team, PHSKC’s medication for opioid use disorder clinic, and King County-based harm reduction organizations. All those involved in recruitment will be trained to prioritize prospective participants’ health and safety. Planned enrollment for Aim 1 is shown in Table 2.

**Table 2:** Aim 1 enrollment by race/ethnicity.

Racial categories	Ethnic categories				Total
	Not Hispanic or Latino		Hispanic or Latino		
	Female	Male	Female	Male	
Aim 1: prospective cohort of overdose survivors					
American Indian/Alaska Native	15	26	0	0	41
Asian	8	16	0	0	24
Native Hawaiian or Other Pacific Islander	7	12	0	0	19
Black or African American	40	80	8	15	143
White	50	100	40	60	250
More than one race	0	0	8	15	23
Total	120	234	56	90	500

During recruitment, potential participants will be able to respond to the study by calling or texting the study phone line printed on study flyers, emailing the study email address, or presenting in person at a data collection site. Prior to the screening process, the study team will ensure that participants understand that the opportunity to participate in the study will be offered independent of any care and after all medical needs have been met (40). The study team will obtain non-written consent over the phone or in person to ask eligibility questions. Study team members will then schedule a time for participants who are eligible for the study to complete the informed consent process at the time and place of their preference. Regardless of eligibility status, participants who are screened will receive an honorarium totaling $10. The compensation amounts for every part of the study were established in collaboration with the community-advisory board to reflect an appropriate, non-compelling, and respectful acknowledgement of participants’ time and the potential risks of the study.

In the informed consent process, the research team members will provide participants with written and verbal information about the study’s objectives, risks, benefits, and how their data will be used (41). The study team will then ask participants if they have any questions and provide answers to the questions they may have. To ensure participants have a clear understanding of study involvement, a member of the study team will ask participants questions about the study. Afterward, participants will provide written informed consent. Participants who are unable to provide informed consent (e.g., due to distress, intoxication, or other impairment) will not be enrolled. Alongside consent forms, Health Insurance Portability and Accountability Act (HIPAA) authorizations will be obtained before any personal health information is accessed, obtained and/or linked to study data. As part of this process, participants will be informed that participation is fully voluntary, and that their decision regarding participation will in no way affect their access to services or the quality of care they receive from King County EMS and other community services. Participants who choose to participate in the prospective cohort study will receive a total of $50 at each time point.

### Aim 1: working toward ongoing participant engagement

ORCID will engage in promising practices and those co-developed with READU to ensure ongoing engagement throughout Aim 1 (42). The engagement strategies are focused particularly on mechanisms to (1) increase ability to maintain ongoing communication with participants, (2) increase visible reminders about the study and bidirectional knowledge sharing about the study in communities of PWUD in King County, and (3) ensure study participation is enjoyable, positive, and easy for participants. Detailed tasks connected to each of the three mechanisms are provided in the Supplementary material on ORCID study engagement strategies.

### Aim 1: data collection and study measures

Participants who have interacted with teams implementing EMS-based interventions will be asked about their experiences with those interactions and their subsequent health outcomes at baseline, 6-, and 12-month follow-ups. Primary outcome measures include discrimination in (1) EMS interactions measured via an adapted everyday discrimination scale (43) for health care interactions and (2) disparities in post-EMS buprenorphine receipt assessed via self-report and linked Prescription Drug Monitoring Program (PDMP) records. Participants will be asked about their experiences of connections to care after contact with EMS, including whether they were offered follow-up care, if they made a successful connection to care, and their perceived satisfaction with the care received. Perceptions of EMS care will be assessed via the emergency nursing-care patient satisfaction scale (ENPSS-21) (44), and perceptions of stigma from EMS providers will be measured via the medical provider stigma experiences by people who use drugs scale (MPS-PWUD) (45). Internal consistency of adapted scales will be assessed using Cronbach’s alpha, and additional validity checks (e.g., test–retest reliability) will be conducted to further evaluate scale performance within the EMS context (46).

To assess access to enhanced services and resources, participants will be asked to describe their EMS encounter and resources offered on scene, including leave-behind naloxone, fentanyl test strips, and warm hand-offs to mobile integrated health teams or PHSKC overdose response teams. Long-term outcomes, including post-EMS buprenorphine receipt, repeat overdose, and pre-EMS naloxone administration for repeat overdose, will be assessed via linked PDMP records and self-report. Additional demographic characteristics will be collected, including age, gender, housing status, income level, employment status, self-reported drug use frequency and severity, criminal justice system involvement, and screening for mental health and substance use disorders.

Possible covariates to be included are the number of hospitalizations, emergency room visits, and jail visits. Demographic characteristics such as age, gender, housing status, race/ethnicity, income level, education level, and employment status will also be collected. Aim 1 study measures are included in Table 3, and the provisionary study instrument is included as a Supplementary material.

**Table 3:** Measures for Aim 1, prospective cohort of overdose survivors.

Variable type (Aim 1)	Construct	Operationalization	Potential data source	CFIR construct
Primary dependent variable(s)	Perceptions of EMS trauma-informed care	Emergency nursing-care patient satisfaction scale (ENPSS-21)	Self-report	Characteristics of individuals
Primary dependent variable(s)	Perceptions of stigma from EMS providers	Medical Provider Stigma Experiences by People Who Use Drugs (MPS-PWUD)	Self-report	Characteristics of individuals
Primary dependent variable(s)	Discrimination in EMS interactions	Discrimination in health care measure via everyday discrimination scale	Self-report	Characteristics of individuals
Primary dependent variable(s)	Post-EMS buprenorphine receipt or other MOUD	Receipt of buprenorphine or other MOUD (Y/N)	Self-report Prescription drug monitoring program	Long-term outcomes
	Connections to care	Offered follow-up care (Y/N) Made a successful connection to care (Y/N) Perceived satisfaction with the care they received Hand-off to PHSKC overdose response team/MIH team Hand-off to MIH team	Self-report EMS Record Management System (RMS)	Short-term outcomes
Intervention fidelity	EMS buprenorphine administration	Did patient experience withdrawal after overdose? Were they offered buprenorphine by EMS? EMS buprenorphine administration (y/n) defined as a buprenorphine prescription given any time on scene for patients Was buprenorphine helpful in alleviating symptoms? Was buprenorphine helpful in improving the encounter/experience overall?	Self-report	
	Patient decisions to accept transport	Transport: (1) yes – to ER; (2) yes – to alternative destination; (3) no	Self-report EMS RMS	Short term outcome
	Resources given to patient or bystander	Resources given to patient or bystander (y/n), where resources include leave-behind naloxone or fentanyl test strips	Self-report EMS RMS	
Moderator	Minoritized racial identity	Minoritized racial identity will be a 3-level variable measuring Black, non-Black Hispanic/Latinx, and non-Hispanic White racial/ethnic identity	Self-report Secondary data	
	Minoritized racial identity (expanded)	Additionally include AI/AN, Native Hawaiian/Other Pacific Islander, Asian	Self-report Secondary data	
Covariates	Number of hospitalizations	# of hospitalizations	Medicaid/Medicare	
	Number of emergency room visits	# of visits	Medicaid/Medicare	
	Number of jail visits (or criminal justice system involvement yes/no)	(# of visits or criminal justice system involvement yes/no)		
	Mortality	Death (yes/no)	Vital statistics	
	Other possible covariates: SUD treatment/behavioral health encounters; screening for SUD and psychiatric disorders; or self-reported rug use frequency and severity			
Demographic characteristics	Age	Age (years)	Self-report Secondary data	
	Gender	Gender identity	Self-report Secondary data	
	Housing status	Housing status	Self-report	
	Income level	Weekly and monthly income; personal and household yearly income	Self-report	
	Education level	Highest grade/degree completed	Self-report	
	Employment status	Current sources of income and job type	Self-report	

### Aim 1: data analysis

The study team estimates that *n* = 500 overdose survivors (*n* = 250 white and *n* = 250 with a minoritized racial/ethnic identity) will consent for the cohort study, with a loss to follow-up of 20% by first follow-up. Assuming that 15% of survivors already have access to buprenorphine treatment, the prospective cohort study will have 80% power to detect a change of 5% in the minimum difference in proportion having linkage to care for buprenorphine treatment (20%) between the minoritized racial groups and the white group (47, 48). This sample size supports subgroup analyses across three racial/ethnic groups (Black, Hispanic/Latinx, AI/AN) and White participants, with stratified analyses supported by our purposive sampling strategy. We have also incorporated false discovery rate (FDR) correction to address multiple comparisons.

Participant survey data will be used to assess differences in the EMS system changes’ implementation characteristics at baseline by race/ethnicity. Analyses will compare minoritized racial/ethnic groups and the white group participants. Participant baseline demographics will be evaluated for group differences. The primary outcome measures will be discrimination in EMS interactions and disparities in post-EMS buprenorphine receipt. Secondary outcome measures will include experiences of connections to care, access to enhanced services and resources, and experiences of the care provided. Exploratory outcomes will include short-term outcomes of likelihood of connection to a response team or mobile integrated health (MIH) team, repeat overdose, and pre-EMS naloxone administration for repeat overdose.

Initial analyses will use descriptive statistics, chi-square tests, and correlational analysis depending on the distributional characteristics of the particular outcomes considered (49). Given the purposive nature of the data collection, we will compare available demographic and clinical characteristics between participants and non-participants using linked secondary EMS data. Sensitivity analyses including inverse probability and propensity score methods may be employed to improve the representativeness of the cohort. To study the outcomes collected at multiple time points, the proposed estimation will use generalized estimating equations with an exchangeable working correlation matrix (50). Robust standard error will be performed to obtain valid test results of correlated outcome data (51). A 5% significance level will be used. Primary data collection at three time points with linkage to multiple systems will additionally allow us to compare data collected directly from participants with data available in administrative systems to better understand data quality issues in analyses based on administrative system data.

### Aim 2: study sample and recruitment

In Aim 2, we will conduct qualitative interviews focused on “centering the margins.” Recruitment will use the same inclusion criteria as Aim 1, with the added criteria for having a minoritized racial/ethnic identity—in line with PHCRP’s emphasis on amplifying the voices of those most impacted by structural inequities. Participants will be sampled utilizing stratified purposive sampling among participants meeting the initial criteria based on racial/ethnic identity and will be recruited on a rolling basis within the prospective cohort (Aim 1) until the targets for the interviews are met or until saturation has been reached (anticipated *n* = 20 Black, *n* = 20 Hispanic/Latinx, *n* = 20 AI/AN individuals). Prospective participants who meet the eligibility criteria will be offered the opportunity to complete an additional qualitative research interview. If they choose to participate in both the quantitative and qualitative data collection, participants will be interviewed after quantitative survey completion and will receive an additional incentive.

Recognizing that consent is an ongoing process, and that participants may change their minds about their interest in being interviewed, the study team will ask all participants to provide verbal and written consent prior to all interviews (52). Additionally, in the consent-seeking process for both the screening and for actual study participation, a member of the study team will address the private and sensitive nature of the questions with participants, let them know that sensitive topics will be discussed, and remind them that they can refuse to answer any questions that they do not want to answer and can withdraw from the study at any time. The study team will provide an honorarium totaling to $100 for participants who participate in any portion of the interview. Throughout Aim 2, the study team will consult the CAB to ensure information presented in the consent process is what a reasonable member of the subject population would want to know (53).

### Aim 2: study measures

Trained interviewers will conduct interviews in private rooms at data collection sites expected to take approximately 30–60 min to complete. Interview questions will be designed to deeply understand experiences of connections to care after contact with EMS, experiences of racial discrimination in contact with EMS, experiences of receiving EMS services and resources, and experiences of trauma-informed care in contact with EMS. The interview guide is included as Supplementary material.

### Aim 2: data analysis and thematic saturation

All interviews will be recorded and transcribed. Interview transcripts will be independently coded by two team members using iterative and team-based thematic analysis beginning with data familiarization, recording initial insights and impressions, and creating a list of preliminary codes from the text itself (54, 55). The coders will first record their initial insights and impressions and create preliminary codes to capture concepts that emerged directly from participant narratives. These codes will be compiled into a preliminary codebook that is developed iteratively. Codes will be defined and arranged into a tree structure and used for line-by-line coding of individual transcripts. The codebook will be treated as an open, living document, and codes will be added and redefined as new themes emerge from the data. Coders will discuss decisions to add codes at least weekly, and earlier transcripts will be revisited when new codes are added to ensure uniform application of codes. Frequent discussion and consensus between coders will ensure inter-coder reliability. Inter-coder reliability will be formally assessed and reported using Cohen’s K and/or the intraclass correlation coefficient.

The overall analytic process employs a combination of deductive and inductive content analysis in which themes are identified based on the interview guide, codes, and existing literature and theory (i.e., PHCRP and CFIR), and then also added during analysis as identified (56). Using principles of co-design, the study team will engage our CAB Action Arm in qualitative analysis, ensuring that their lived experience contributes to framing and interpretation of findings. Involvement of CAB members will follow a participatory process, preparing and including team members in co-analysis of qualitative data as our team has done previously (57). The investigative team, in partnership with the CAB Action Arm, will review templated data to finalize themes, check conclusions against the data, and identify prototypic examples within each theme for presentation. Additionally, Synthesized Member Checking will be conducted, inviting participants to provide feedback on the summary of findings and incorporating their feedback to enhance accuracy and validity when they are contacted for follow-up interviews for Aim 1 (58). Consistent with PHCRP, Aim 2 centers the experiences and perspectives of minoritized racial/ethnic patients rather than contrasting them with patients from more dominant groups. By doing so, Aim 2 methods avoid the common pattern of reinforcing majority experiences as the norm and minority experiences as deviations or deficits (59).

Thematic saturation will be evaluated in the context of several dimensions of information power, including the specificity of the sample and the quality of the dialog (60). The Aim 2 sample includes individuals who meet highly specific criteria: they have experienced an overdose within the past 6 months, identify as having a minoritized racial/ethnic identity, and had their overdose responded to by EMS. Thus, we believe that the participant characteristics are highly specific and increase the information power. Furthermore, the participatory nature of our study design is expected to enable strong rapport and clear communication between participants and researchers. This co-constructed dialog will enhance the relevance of the interviews and contribute to high information power. Finally, we will implement a saturation tracking grid to document the emergence of new codes and calculate the new-code rate by interview, which will allow us to systematically assess when we are approaching thematic situation.

### Aim 3: study sample

Aim 3 will use secondary data obtained from the EMS Division of PHSKC’s records management system (RMS) that assimilates data from four 9-1-1 dispatch centers, 28 basic life support fire departments, 5 advanced life support paramedic groups, and over 20 hospitals via a health data exchange. Additionally, data beyond the EMS encounter is pulled into EMS RMS through the Comprehensive Hospital Abstract Reporting System (CHARS). All data stored on secure network servers are managed by King County Information Technology (KCIT) and meet all regulations and requirements for PHSKC as a HIPAA-compliant entity. Data are collected and processed daily with routine quality assurance checks. The study sample for Aim 3 will be all patients seen by King County’s EMS division between January 2021–December 2026 for probable overdose. Per prior studies utilizing King County EMS data (12), probable overdose will be defined as having met at least five or more of the following criteria, or four criteria including the receipt of naloxone or documentation of pinpoint pupils: (1) primary or secondary impressions indicative of opioid overdose; (2) “opioid” key words; (3) “overdose” key words; (4) respiratory rate < 11 breaths per minute OR “decreased respirations” key words; (5) Glasgow Coma Scale < 15 OR “decreased level of consciousness” key words; (6) naloxone listed as a discrete EMS-administered medication OR indicated in the free text narrative; (7) “pinpoint pupils” key words, and (8) “drug use” key words. Probable overdoses characterized in this way have high sensitivity, as 90% of KC-EMS incidents labeled as probable overdose were actual overdoses (61). The sample size for Aim 3 is expected to be 10,800 unduplicated EMS encounters for acute opioid overdose, with 5,700 patients contacted by EMS-OPP trained teams (i.e., total *n* = 10,800, with *n* = 5,700 receiving the intervention across the study timeline).

### Aim 3: study measures

The primary outcome for Aim 3 will be patient connection to follow-up care for access to buprenorphine. Additional measures will include patient decisions to accept transport, transport destination, resources given to patient or bystander, repeat overdose events, pre-EMS naloxone administration for repeat overdose events, post-EMS buprenorphine administration, subsequent emergency department visit, and subsequent hospitalization.

The exposure variable will be overdose events attended by an EMS team trained on the EMS-based intervention (with trauma-informed care EMS training as the focal system change), with the number of trained teams increasing over time. Other EMS system changes will be included as control variables.

Minoritized racial/ethnic identity will be used as a primary moderator, measured as a 3-level variable (Black, non-Black Hispanic/Latinx, and non-Hispanic White racial/ethnic identity) based on EMS record documentation. Additional individual demographic characteristics, including age, sex, and housing status, and neighborhood-level variables derived from the location of the overdose response will also be included.

### Aim 3: data analysis

The sample size of the secondary data analysis (Aim 3) is estimated to be 10,800 patients with a probable overdose seen by King County’s EMS division between January 2021–December 2026 for probable overdose. The study team will examine all patients with probable overdose in EMS encounters in monthly increments over the 12 months prior to the implementation of the trauma-informed care training for EMS and 12 months after the system changes are implemented resulting in 24 time periods (months) under investigation. Based on simulation-based power calculations for interrupted time series analyses, assuming that 20% of the intervention’s effect is from a level change and 80% is from a trend change, we estimate that for our fixed sample size of 24 (12 time points pre- and post-intervention/system changes), we will have >80% power to detect a moderate (i.e., 1.0) and large (i.e., 2.0) effect of the system changes (62). Power will be decreased if there is autocorrelation present and if there is an imbalance in study time periods pre and post intervention. To assess the robustness of our interrupted time series (ITS) design, we conducted a sensitivity analysis to estimate the minimum detectable effect size under varying levels of autocorrelation (*ρ* = 0.1, 0.3, 0.5). Assuming 24 monthly time points (12 pre- and 12 post-intervention), we found that the effective sample size decreased substantially with increasing autocorrelation, resulting in larger minimum detectable effect size values. Specifically, Cohen’s h was 0.126 for ρ = 0.1, 0.156 for ρ = 0.3, and 0.198 for ρ = 0.5. These findings underscore the importance of accounting for autocorrelation in our models. Accordingly, we will test for autocorrelation using Durbin-Watson and Ljung-Box tests and incorporate autoregressive terms or generalized estimating equations with appropriate correlation structures to ensure valid inference. We will also interpret null findings in light of these power constraints.

Primary analysis will examine whether the EMS system changes’ impact differed between Black compared to non-Hispanic White patients, and Hispanic/Latinx compared to non-Hispanic White patients (in a separate model), using multiple group interrupted time series. We will use segmented logistic regression models fit using generalized estimating equations to account for clustering of patients within geographic regions, with autoregressive correlation structure (63). To strengthen causal inference, we will pre-specify model parameters such as the intervention start date, the functional form of time (i.e., continuous monthly time), and the lag structure of the model. Model selection will be guided by fit indices, such as the Akaike Information Criterion and Bayesian Information Criterion, alongside residual diagnostics. We will also assess for potential seasonality by visually inspecting time series plots and including monthly indicator variables if patterns are observed. We will use multiple imputation by chained equations (MICE) to address missingness in covariates and outcomes, assuming data are missing at random. We will also conduct sensitivity analyses to assess the robustness of our findings to different missing data assumptions.

Models will estimate change in the level and trend of the log odds of the outcome 12 months prior to and following the implementation date of the EMS-based interventions, adjusting for the pre-implementation trend in outcome. Models will include interaction terms to compare changes between Black or Hispanic/Latinx and White patients. We will examine two estimates: (1) difference across race/ethnicity in the level of the log odds of the outcome immediately following implementation and (2) difference across race/ethnicity in the slope (trend) of the log odds of the outcome after implementation compared with pre-implementation.

### Data linkage and integrated analyses

Datasets from both the prospective cohort (Aim 1) and the secondary data analysis (Aim 3) will be linked, via a unique participant ID, with data systems across multiple institutions including vital statistics, King County EMS, King County Jail, Medical Examiner’s Office, Prescription Drug Monitoring Program, and Medicaid data. This data linkage process has been piloted and successfully completed retrospectively by PHSKC, with regular quality assurance checks, thus facilitating subsequent linkage processes (12). As part of the informed consent process for the prospective cohort (Aim 1), participants will be asked whether they would like to opt out of the data linkage process. The data governance and de-identification procedures will be aligned with the requirements put forth by both the University of Washington Human Subjects Division Institutional Review Board and the Washington State Institutional Review Board.

For integrated analyses, quantitative and qualitative data from Aims 1–3 will be analyzed independently and then synthesized. This will allow findings to be shared as they emerge with key study stakeholders, who can apply the information into their roll-out of EMS system changes. Since the purpose is triangulation, we will conduct integration after the point of analysis to develop a holistic understanding of the impact of the structural interventions in the EMS system on racial disparities in health outcomes. Results will be summarized across approach and source, and additional insights gained from integration will be added in a joint analysis section of final reporting documents.

## Discussion

This implementation-effectiveness study capitalizes on natural experimental conditions to employ a concurrent triangulation mixed-methods design, guided by CFIR and PHCRP, to evaluate mechanisms driving existing disparities in overdose outcomes and prepare for future surveillance of emerging inequities. Through a community-partnered evaluation of EMS systems changes in King County, study findings will inform how EMS systems can address the opioid epidemic and associated disparities.

### Advantages and innovations

The ORCID study addresses existing limitations in surveillance systems by comparing EMS-recorded race/ethnicity data with self-reported measures and collecting new data on mechanisms of action within EMS and a range of health outcomes not previously captured (64–66). As the first study of its kind to establish a longitudinal cohort of non-fatal overdose survivors, this work has the potential to inform policy decisions regarding EMS changes. Specifically, this study allows for in-depth understanding of how EMS system changes are differentially experienced by minoritized racial/ethnic groups, and the longitudinal design will track both short- and long-term overdose-related outcomes. Moreover, through integration of data across multiple systems, this study will be conducive for the continued monitoring of overdose outcomes and associated disparities at the individual and population levels far past the study end dates.

### Embodiment of community engagement and support

Importantly, in championing a participatory community engagement approach throughout the entire study, ORCID includes multi-partner ownership of the research process by design and ensures that lived experience is centered in an iterative and emergent manner (67, 68). In centering the voices of individuals most impacted by this research, the study approach incorporates both a traditional Community Advisory Board and an innovative Action Arm, comprised of team members who actively collaborate in shaping study tools, develop a peer support philosophy, and contribute to data collection. Wielding open dialog, trust-building, and reciprocity, the Action Arm creates a space for mutual learning and critical reflexivity. READU members contribute their lived and living experience, ensuring that research goals and methodologies remain grounded in the reality of the communities served. This approach enhances the relevance and impact of the study and challenges traditional research hierarchies by valuing experiential knowledge alongside academic expertise. By building this research project together, ORCID strives to set a new standard for equity-driven research that prioritizes meaningful, actionable, and community-informed outcomes.

Through a redistribution of power and decision-making across the research team, ORCID remains intentionally flexible in the research process to allow for adaptive decision-making—ensuring that community partners, including individuals with lived and living experience, have genuine influence over study activities. By remaining open to shifting timelines, evolving methodologies, and incorporating feedback in real time, the study team environment seeks to be one where all members—regardless of academic status or institutional affiliation—feel valued and heard. This adaptability fosters a culture of co-learning and reciprocity, where traditional hierarchies are subverted when possible, and diverse perspectives are not only welcomed but also drive the direction of the research.

To prioritize participant support, the ORCID study design integrates peer support specialists to broaden the scope of the CBPR approach and enhance the mutuality of the data collection processes—extending benefits to study participants (69). Peer support as a cornerstone of study team design validates lived and living experiences as forms of expertise that directly contribute to the delivery of quality healthcare for individuals facing challenges related to substance use, marginalization, and systemic inequities. This approach acknowledges the unique ability of peer support specialists to foster trust, build meaningful connections with participants through shared experiences, and meet participants where they are at.

### Team diversity and reflexivity practice

Similarly, ORCID is further strengthened by an intentional commitment to reflexivity, ensuring that research processes remain accountable to both scientific rigor and the lived experience of those impacted by racial inequities and overdose disparities (70, 71). Through weekly meetings within the community-based research team, diverse perspectives are integrated and extend beyond traditional academic frameworks to foster a more participatory and community-driven research approach. These discussions not only enhance the study design but also challenge academic researchers to remain critical of their own positionality by recognizing and responding to the ways in which power, privilege, and lived and living experience shape the research process.

Underscoring ORCID’s commitment to diversity, the study team is comprised of individuals with diverse backgrounds and expertise to ensure a thoughtful approach to positionality and reflexivity (72). Members represent historically underrepresented groups in National Institutes of Health funding—including underrepresented racial and ethnic minority-individuals, women and non-binary individuals, and those with lived or living experience of drug use. At varied career stages, from those new to research to seasoned experts, alongside representation from research-intensive universities, community-based organizations, local health departments, and EMS organizations, diversity is a team value.

### Study limitations

A primary challenge is the iterative nature of EMS system changes, which complicates consistent data collection. The approval and implementation of new overdose crisis care centers and the unexpected introduction of EMT-administered buprenorphine in Seattle during the study period highlight the evolving nature of the intervention landscape. This fluidity requires continuous engagement with EMS partners and adaptability in research methodologies. Unmeasured concurrent changes may introduce confounding and affect the comparability of measures between baseline and follow-up periods. To account for known concurrent EMS system changes, the study team will work closely with local public health agencies to develop fidelity measures including the percentage of teams trained in planned changes, adherence to implemented protocols, and documentation of other relevant system-level initiatives occurring during the study period. These fidelity metrics will inform sensitivity analyses and help contextualize the observed outcomes in the study. Additionally, the interrupted time series design assumes stable pre-intervention trends that may not hold given the highly dynamic nature of the overdose landscape in King County. For example, the broader trend of overdose rates in King County demonstrated a peak of overdoses in 2022–2023 and declines in 2024 (1). We acknowledge that this may limit the causal inference of the analyses and hope to incorporate alternative quasi-experimental methods such as synthetic controls to better account for these fluctuations.

Another limitation is the non-random nature of the purposive sample, which introduces potential biases associated with self-selection (73). To reduce selection bias, we will attempt to conduct sensitivity analyses including comparisons of participants’ and non-participants’ demographic characteristics, as well as implementation of inverse probability weighting or propensity score methods to extend the generalizability of findings beyond the study participants. Given that many overdose-related measures in the primary data collection are self-reported, there is concern around recall bias. However, we plan to mitigate these challenges by linking self-reported, participant data with administrative records to reduce misclassification and improve the accuracy of overdose-related outcomes. While our community-engaged approach, including peer support specialists, is expected to enhance retention, maintaining longitudinal follow-up with overdose survivors remains a challenge. To address this, the study team has powered the analyses to account for loss-to-follow-up and will intentionally recruit from locations around King County. Furthermore, the use of semi-structured interviews is designed to better capture experiences of discrimination and stigma, subsequent overdose, or use of harm reduction supplies—shaped by an individual’s last overdose experience.

While this study explores the experiences of minoritized racial/ethnic groups following non-fatal overdose and EMS response, there may be potential for unintended consequences stemming from both the research design and the broader EMS system changes under evaluation. Repeated follow-up may risk contributing to participant distress or secondary trauma, particularly among individuals experiencing housing instability, ongoing substance use, or recent overdose. Data collection and outreach efforts may also increase demands on EMS personnel, public health staff, and community partners, potentially diverting resources from other essential services. Well-intentioned interventions (e.g., prehospital buprenorphine, outreach follow-up, and cross-system data linkage) could be perceived as intrusive or stigmatizing, especially in communities already subject to heightened surveillance and discrimination. If post-overdose interventions or research participation are seen as coercive or misaligned with individual needs, they may also further erode trust in EMS and healthcare systems. To address these risks, the study is grounded in community-engaged research principles and incorporates qualitative methods specifically designed to explore experiences of stigma, care quality, and system-level trust, with the goal of informing more equitable and patient-centered implementation strategies.

Reliance on secondary data sources also introduces limitations in available race/ethnicity data, which may not properly reflect participants’ identities. Additionally, linking prescription drug monitoring data to assess subsequent buprenorphine prescriptions presents challenges, as some prescriptions—particularly injectable buprenorphine—may not always appear in these databases. Methadone treatment is also absent from this data, requiring additional linkage to Medicaid records. To mitigate these gaps, the study team is planning on collecting self-reported data on medication receipt. Similar challenges exist in tracking subsequent overdose events, which may be underestimated due to data linkage limitations. Finally, the study is unable to track care connections occurring outside EMS and emergency departments, likely resulting in an underestimation of the intervention’s true effects. However, any resulting bias is expected to be conservative and lean toward the null hypothesis, which is preferable to inflating EMS system change impact estimates.

### Ethics and dissemination

The ORCID study is strongly committed to ethical practices and meaningful dissemination to community partners utilizing a combination of CBPR and Integrated Knowledge Translation (ITT) (74, 75). ITT posits a collaborative process that emphasizes partnerships between researchers and the people for whom the research is ultimately meant to be of use (“knowledge users”). It is a dynamic, ongoing process of exchanging, disseminating, and applying knowledge between partners, allowing research to be responsive to evolving needs while fostering a deeper partnership between researchers and community members.

As part of this process, the study team will develop and disseminate knowledge that is co-produced alongside individuals with lived and living experience of drug use, representative, and actionable. Engaging the community and community-based research team ensures that co-created knowledge reflects lived realities of community partners, while collaboration with partners like EMS providers and PHSKC ensures that our research recommendations are feasible and acceptable. This multidirectional exchange of knowledge creates a holistic and practical research model that centers the needs of PWUD, ensuring that our findings are meaningful and do not inadvertently harm the communities we work with.

Dissemination will involve both practice or community-based products and traditional research products such as scientific publications. Practice products include policy briefs or summary documents for EMS providers and our practice partners at PHSKC. Community-oriented products such as a community zine and art posters will also be developed to build study awareness and share research results with the PWUD community. To support policy change at the local level, we will leverage our team’s ongoing advisory role with PHSKC and continue to provide technical assistance related to implementing EMS strategies to reduce inequities in overdose response. At the state level, we will collaborate with partners such as the Center for Evidence-based Addiction Treatment (CEADU) and the Voices of Community Activists and Leaders – Washington (VOCAL-WA) (76), both of which are actively involved in substance use policy advocacy in Washington state. At the national level, we hope that our evaluation of EMS system changes in King County—widely recognized as having one of the best EMS systems in the world (77)—positions us to contribute to national discourse around evidence-based EMS interventions to reduce racialized inequities in overdose outcomes.

Throughout dissemination, all developed products will be subject to Synthesized Member Checking (58), which invites participants to provide feedback on findings to enhance accuracy and validity. This work will provide data for subsequent funding applications, sustaining and expanding the longitudinal cohort we plan to develop, and importantly extending our commitment to this pressing public health issue by uplifting the voice of historically marginalized communities.

## Conclusion

This study proposes a comprehensive and innovative approach to understanding and addressing racial disparities in overdose outcomes through the evaluation of EMS system changes in King County. By employing advanced implementation science guided by the PHCRP and centered around community partnerships, the ORCID study aims to provide valuable insights into the mechanisms driving existing inequities and prepare for future surveillance of racial disparities in overdose outcomes. The study’s strengths lie in its commitment to community engagement, integration of peer support, and emphasis on diversity and reflexivity within the research team. Through the establishment of a longitudinal cohort of non-fatal overdose survivors and the integration of data across multiple systems, this research has the potential to inform policy decisions and improve EMS responses to the opioid epidemic, both within King County and beyond.
