# Supplementary material for: Overdose response centering inequity and diversity study: a protocol for assessing the population-level and equity impact of the emergency medical services system changes using critical race theory
Source: Front Public Health. 2025 Sep 15;13:1629518. doi: 10.3389/fpubh.2025.1629518 (PMC12477233; doi:10.3389/fpubh.2025.1629518)
Supplement: Supplementary file 1 [file Data_Sheet_1.PDF]

Supplemental Material: ORCID Study Engagement Strategies

Mechanisms	Tasks
Increase ability to maintain ongoing communication with participants	1. Obtaining permission to contact participants in non-traditional ways (via social media, permission to have a second point of contact beyond their next of kin such as roommates, friends, case managers), and permission for study staff to be alerted through medical records if a study participant has an EMS or emergency department visit;
	2. Updating contact information at all time-points, giving appointment slips and/or fridge magnets (stating time, place, and contact person for next appointment), reminder calls;
	3. Sending birthday postcards/text messages to facilitate an additional point of contact each year with each participant;
	4. Offering participants the possibility of utilizing the “study buddy” approach used by comparable cohort studies of PWUD where participants are invited to pick a friend or family member to become their “study buddy,” if they would like extra social or familial support to stay engaged in the study. Study buddies and participants will watch a video on the study aims developed by our CAB Action Arm and buddies will receive instruction to remind their participant of follow-up appointments to help encourage retention.
	5. Maintaining a consistent presence and prioritizing community involvement for our study staff will ensure that the importance of retention and reminders will be conveyed regularly in the context of relationship-building with participants.
Increase visible reminders about the study and bi-directional knowledge sharing about the study in the community of PWUD in King County	6. Having engaging visual study materials (designed by our CAB Action Arm) that participants see regularly to facilitate ongoing engagement such as: stickers with the study logo, posters with study logo and contact information to remind participants of the aims of the study displayed at local treatment clinics and community service organizations;
	7. Having visibility of study personnel via regular physical presence (e.g., the peer support specialist) in community spaces where PWUD frequently visit to offer study updates and ongoing connections to any PWUD who are interested in discussing the study;
	8. Beginning the process of bi-directional knowledge translation in year 1, the study team will facilitate the sharing of knowledge and wisdom from PWUD with other PWUD through the co-creation of issues of a zine that include art, stories, and submissions from PWUD.
Ensure study participation is enjoyable, positive, and easy for participants	9. Peer support specialists will build relationships with participants during their study visits, debriefing on the study as desired, making the experience of participating in the research study a grounding experience and something that participants look forward to and want to continue participating in;
	10. Providing multiple options for suitable locations for completing study visits, giving participants the ability to choose their preferred location from a range of different university-affiliated facilities and community service locations (e.g., medical center, crisis stabilization center, PHSKC buprenorphine clinic, etc.);
	11. Making research spaces warm and welcoming: having snacks and drinks available to participants, having space for participants to securely store their possessions during study visits;
	12. Sending participants a thank you card/text message the week after their study visit to let them know how much their participation is valued and appreciated and to offer them the opportunity to share anonymous feedback regarding ways to improve comfort and ease of participation;
	13. In the video about study aims designed by the CAB action arm referenced above, the study team will share details about the motivation and urgent need for the study from the perspectives of our CAB and community partners; extending sincere, personal gratitude to participants for their contributions to science and to improving overdose care in King County.
