# Supplementary material for: Overdose response centering inequity and diversity study: a protocol for assessing the population-level and equity impact of the emergency medical services system changes using critical race theory
Source: Front Public Health. 2025 Sep 15;13:1629518. doi: 10.3389/fpubh.2025.1629518 (PMC12477233; doi:10.3389/fpubh.2025.1629518)
Supplement: Supplementary file 2 [file Data_Sheet_2.PDF]

Baseline Survey

READ: Thanks again for agreeing to take part in this study. Throughout this interview, if we get off track, I might ask us to come back to the survey questions. We're in this together and I don't mind if we get off track, but I want to be mindful of your time and the other folks who have signed up to do the survey today.

So to begin, I'm going to ask you some questions about your experiences with Emergency Medical Services, or EMS. To reiterate, for the purposes of this study, EMS is any professional that responded to or showed up at your overdose. And by overdose, I mean if you passed out, turned blue, or stopped breathing from using drugs.

HIDDEN, AUTOMATIC: Start time where respondent starts answering questions

____

O1. How many times have you overdosed in the past 6 months? Please give your best estimate.

____

O2. When was your most recent overdose that EMS responded to? Please tell me the date and time. We have a calendar here that you can use.

____
(If they do not remember the exact date and time you can ask for an estimate)

Refer to pages 3 & 4 of the Interview Booklet for the next questions.

O3. Where did this overdose occur?

____
(Insert street address or intersection, or description of physical or geographic location)

O3A. Was this an estimate or exact location?

- ◯ Estimate
- ◯ Exact location
- ◯ Unsure
- ◯ Prefer not to answer

Refer to Page 5 for the next questions about different types of first responders, including EMS. I'm going to briefly review each.

As you may know, paramedics provide on-scene advanced life support, arrive in ambulances, and can administer medications, whereas firefighters provide basic life support and assist with stabilization efforts such as administering CPR and intranasal naloxone but typically are not medical professionals.

Mental health providers, including social workers, might also arrive as part of a co-response or mobile integrated health team. Police are members of law enforcement who can administer intranasal naloxone and assist with transportation but are not members of the medical team.

Bystanders might also be present and may have offered support to you.

E1. Do you remember what kind of first responders showed up at your overdose (for example, paramedics, firefighters, mental health providers, police, or bystanders)?	◯ No<br>◯ Yes<br>◯ Don't Know<br>◯ Prefer not to Answer
E1a. How many paramedics showed up to your overdose? Please give your best estimate.	__________________________
E1b. How many firefighters showed up to your overdose? Please give your best estimate.	__________________________
E1c. How many mental health providers showed up to your overdose? Please give your best estimate.	__________________________
E1d. How many police officers showed up to your overdose? Please give your best estimate.	__________________________
E1e. How many bystanders were present at your overdose? Please give your best estimate.	__________________________
E1f. How many unknown EMS providers responded to your overdose?	__________________________
E1g. Did anyone who does not fit into any of these categories respond to your overdose?	◯ No<br>◯ Yes<br>◯ Unsure<br>◯ Prefer not to answer
E1h. For those who did not fall under any of the above categories, how many were present or showed up to your overdose?	__________________________
So in total, [___] first responders showed up to your overdose?	__________________________

E2. Refer to Page 6 of the Interview Booklet for the next question. On a scale from 1 to 5, rate how much interaction you had with each type of individual (with 1 being almost no interaction and 5 being the highest level of interaction). If you don't know exactly what type of provider you were interacting with, that's okay, just take your best guess.

	Almost none (1)	A little bit of interaction (2)	A moderate level of interaction (3)	Lots of interaction (4)	Interacted with me the most (5)	No interaction	Prefer not to Answer
Paramedics/ALS	○	○	○	○	○	○	○
Firefighters/BLS	○	○	○	○	○	○	○
Mental health providers	○	○	○	○	○	○	○
Police	○	○	○	○	○	○	○
Bystanders	○	○	○	○	○	○	○
Unknown EMS Providers	○	○	○	○	○	○	○
Others who do not fall under above categories	○	○	○	○	○	○	○

READ: Now we have some questions about how you were treated by the EMS providers who responded to your overdose.

E3. Refer to Page 7 of the Interview Booklet for the next question. In the last question, we asked about the level of interaction you had with different types of first responders. In this next question, please consider how you were treated by the EMS providers who responded to your most recent overdose. We are not asking about specific type of providers, so please give us your general impression of the experience, as best as you can remember. On a scale from 1 to 5, with 1 being strongly disagree and 5 being strongly agree, rate to what extent each of the following is true.

	Strongly disagree (1)	Disagree (2)	Neither agree nor disagree (3)	Agree (4)	Strongly agree (5)	Don't Know	Refuse to Answer
EMS provided me with a physical space that felt comfortable and safe (e.g., private area, ensured privacy from bystanders & other responders, and provided an adequate and safe distance between me and the providers).	○	○	○	○	○	○	○
EMS initially approached me in a non-judgmental and compassionate way.	○	○	○	○	○	○	○
EMS clarified my situation and need for services.	○	○	○	○	○	○	○
EMS communicated openly (e.g., provided all relevant information, being transparent) with me.	○	○	○	○	○	○	○
EMS listened to me (e.g., paid attention to what I said, didn't interrupt me) and made eye contact with me when they were interacting with me.	○	○	○	○	○	○	○
EMS allowed and encouraged expression of my feelings (even negative emotions) without judgment (e.g. didn't assert judgement on me).	○	○	○	○	○	○	○
EMS made me feel emotionally safe by providing reassurance and validating my experiences.	○	○	○	○	○	○	○
EMS used a professional tone to convey genuine concern.	○	○	○	○	○	○	○

EMS explained things in a way I could understand (e.g., used plain language without jargon).	○	○	○	○	○	○	○
EMS involved me in decisions about my care.	○	○	○	○	○	○	○
EMS checked that I understood the information they were providing to me	○	○	○	○	○	○	○
EMS recognized power imbalances (e.g., acknowledged that I know my own life best).	○	○	○	○	○	○	○
EMS provided encouragement, used kind language, and avoided being critical.	○	○	○	○	○	○	○
EMS used statements like, "It's your decision," and "It's not for me to decide" that made me feel like I had autonomy in my care decisions.	○	○	○	○	○	○	○

E3. We are going to go through the same list of questions once more. Refer to Page 8 of the Interview Booklet this time. So, regardless of whether the following happened to you, please rate how important each of the following is to you during an interaction with EMS for an overdose.

	Not at all important (1)	Slightly important (2)	Moderately important (3)	Important (4)	Very important (5)	Don't Know	Refuse to Answer
EMS provide me with a physical space that feels comfortable and safe (e.g., private area, ensure privacy from bystanders & other responders, and provide an adequate and safe distance between me and the providers).	○	○	○	○	○	○	○
EMS initially approach me in a non-judgmental and compassionate way.	○	○	○	○	○	○	○
EMS clarify my situation and need for services.	○	○	○	○	○	○	○
EMS communicate openly (e.g., provide all relevant information, being transparent) with me.	○	○	○	○	○	○	○
EMS listen to me (e.g., pay attention to what I'm saying, do not interrupt) and make eye contact with me when they are interacting with me.	○	○	○	○	○	○	○
EMS allow and encourage expression of my feelings (even negative emotions) without judgment (e.g. do not assert judgement on me).	○	○	○	○	○	○	○
EMS make me feel emotionally safe by providing reassurance and validating my experiences.	○	○	○	○	○	○	○
EMS use a professional tone to convey genuine concern.	○	○	○	○	○	○	○
EMS explain things in a way I can understand (e.g., use plain language without jargon).	○	○	○	○	○	○	○
EMS involve me in decisions about my care.	○	○	○	○	○	○	○

EMS check that I understand the information they are providing to me	○	○	○	○	○	○	○
EMS recognize power imbalances (e.g., acknowledging that I know my own life best).	○	○	○	○	○	○	○
EMS provide encouragement, use kind language, and avoid being critical.	○	○	○	○	○	○	○
EMS use statements like, "It's your decision," and "It's not for me to decide" that make me feel like I had autonomy in my care decisions.	○	○	○	○	○	○	○

Now we have some different questions about your experience with EMS and first responders.

E4. When I interacted with police after my last overdose experience, I felt the interaction was:

- ◯ Very good
- ◯ Good
- ◯ Neutral
- ◯ Bad
- ◯ Very bad
- ◯ N/A; did not interact with police
- ◯ Prefer not to answer

E5. Refer to Page 9 of the Interview Booklet for the next question. Were you transported anywhere following your overdose?

- ◯ Yes - Emergency Department
- ◯ Yes - Crisis Solutions Center / Lane St near Dearborn & Rainier Street
- ◯ Yes - ORCA Center/Morrison on 3rd St
- ◯ Yes - Connections Kirkland crisis response center
- ◯ Yes - Somewhere else
- ◯ No - Transportation was not considered medically necessary
- ◯ No - I declined transportation
- ◯ Don't Know
- ◯ Prefer not to answer

E5a. Where you were you transported following your overdose?

____

E5b. Please explain why you decided to decline transportation to a medical facility following your overdose.

____

The following question is about your experiences with EMS and other medical providers.

E6. For the following questions, refer to Page 7 of the Interview Booklet for the next question. Please consider your most recent overdose interaction with EMS. On a scale of 1 to 5, with 1 being strongly disagree and 5 being strongly agree, rate how much you agree with the following statements.

	Strongly disagree (1)	Disagree (2)	Neither agree nor disagree (3)	Agree (4)	Strongly agree (5)	Don't Know	Prefer not to answer
The EMS provider(s) said critical or insulting things to me about my drug use.	○	○	○	○	○	○	○
The EMS provider(s) made me feel upset by comments they made about my drug use.	○	○	○	○	○	○	○
I felt that I was treated disrespectfully by the EMS provider(s) because of my drug use.	○	○	○	○	○	○	○
I felt that I could not speak freely with the EMS provider(s) about my drug use.	○	○	○	○	○	○	○
I felt that EMS provider(s) did not treat me as nicely as they would have treated a patient who does not use drugs.	○	○	○	○	○	○	○
EMS provider(s) have told me I need to quit using drugs without my asking them.	○	○	○	○	○	○	○
I felt that the EMS provider(s) didn't understand my experience as a person who uses drugs.	○	○	○	○	○	○	○
I did not want to disclose my drug use to the EMS provider(s).	○	○	○	○	○	○	○
I felt that the EMS provider(s) was afraid of me.	○	○	○	○	○	○	○
I felt respected by the EMS provider(s).	○	○	○	○	○	○	○

E6a. READ: You mentioned being transported to the Emergency Department following your most recent overdose interaction with EMS. On a scale of 1 to 5, with 1 being strongly disagree and 5 being strongly agree, rate how much you agree with the following statements.

	Strongly disagree (1)	Disagree (2)	Neither agree nor disagree (3)	Agree (4)	Strongly agree (5)	Don't Know	Prefer not to answer
When I arrived at the Emergency Department, I felt as though I had to try and hide my drug use.	◯	◯	◯	◯	◯	◯	◯
When I went to the Emergency Department, I felt embarrassed and/or ashamed for being a drug user.	◯	◯	◯	◯	◯	◯	◯
I did not feel welcome in the Emergency Department.	◯	◯	◯	◯	◯	◯	◯

E6b: You mentioned being transported to the Crisis Solutions Center (at Lane St near Dearborn & Rainier Street) following your most recent overdose interaction with EMS. On a scale of 1 to 5, with 1 being strongly disagree and 5 being strongly agree, rate how much you agree with the following statements.

	Strongly disagree (1)	Disagree (2)	Neither agree nor disagree (3)	Agree (4)	Strongly agree (5)	Don't Know	Refuse to Answer
When I arrived at the Crisis Solutions Center, I felt as though I had to try and hide my drug use	○	○	○	○	○	○	○
When I went to the Crisis Solutions Center, I felt embarrassed and/or ashamed for being a drug user.	○	○	○	○	○	○	○
I did not feel welcome at the Crisis Solutions Center.	○	○	○	○	○	○	○

E6c. READ: You mentioned being transported to the ORCA Center (at Morrison on 3rd St) following your most recent overdose interaction with EMS. On a scale of 1 to 5, with 1 being strongly disagree and 5 being strongly agree, rate how much you agree with the following statements.

	Strongly disagree (1)	Disagree (2)	Neither agree nor disagree (3)	Agree (4)	Strongly agree (5)	Don't Know	Refuse to Answer
When I arrived at the ORCA Center, I felt as though I had to try and hide my drug use.	○	○	○	○	○	○	○
When I went to the ORCA Center, I felt embarrassed and/or ashamed for being a drug user.	○	○	○	○	○	○	○
I did not feel welcome at the ORCA Center.	○	○	○	○	○	○	○

E6d. READ: You mentioned being transported to Connections Kirkland crisis response center following your most recent overdose interaction with EMS. On a scale of 1 to 5, with 1 being strongly disagree and 5 being strongly agree, rate how much you agree with the following statements.

	Strongly disagree (1)	Disagree (2)	Neither agree nor disagree (3)	Agree (4)	Strongly agree (5)	Don't Know	Refuse to Answer
When I arrived at Connections Kirkland, I felt as though I had to try and hide my drug use.	○	○	○	○	○	○	○
When I went to Connections Kirkland, I felt embarrassed and/or ashamed for being a drug user.	○	○	○	○	○	○	○
I did not feel welcome at Connections Kirkland.	○	○	○	○	○	○	○

E6e. READ: You mentioned being transported somewhere following your most recent overdose interaction with EMS. On a scale of 1 to 5, with 1 being strongly disagree and 5 being strongly agree, rate how much you agree with the following statements.

	Strongly disagree (1)	Disagree (2)	Neither agree nor disagree (3)	Agree (4)	Strongly agree (5)	Don't Know	Refuse to Answer
When I arrived at that place, I felt as though I had to try and hide my drug use.	○	○	○	○	○	○	○
When I went to that place, I felt embarrassed and/or ashamed for being a drug user.	○	○	○	○	○	○	○
I did not feel welcome at that place.	○	○	○	○	○	○	○

Progress check: We've made it through about 25% of our interview questions.

For the next set of questions, you will be asked whether certain things may have happened to you due to your racial or ethnic identity. For these questions, please consider your most recent overdose interaction with EMS. For each item you can respond with never, rarely, sometimes, most of the time, or always. Refer to Page 10 of the Interview Booklet for response options to these questions.

E7. During your most recent overdose interaction with EMS, did any of the following happen to you because of your race or ethnicity?

	Never (1)	Rarely (2)	Sometimes (3)	Most of the time (4)	Always (5)	Don't Know	Prefer not to answer
Due to your race or ethnicity, you felt like the EMS provider(s) were not listening to what you were saying.	◯	◯	◯	◯	◯	◯	◯
Due to your race or ethnicity, you were treated with less respect than other people by the EMS provider(s).	◯	◯	◯	◯	◯	◯	◯
Due to your race or ethnicity, the EMS provider(s) acted as if they thought you were not smart.	◯	◯	◯	◯	◯	◯	◯
Due to your race or ethnicity, the EMS provider(s) acted as if they were afraid of you.	◯	◯	◯	◯	◯	◯	◯

Now we have some questions about your experience with the criminal justice system after your most recent overdose.

C1. Were you arrested or detained during or immediately after your overdose?

◯ No
◯ Yes, arrested
◯ Yes, detained
◯ Don't Know
◯ Prefer not to answer

C2. Was anyone else at the scene arrested or detained during or immediately after your overdose?

◯ No
◯ Yes, arrested
◯ Yes, detained
◯ Don't Know
◯ Prefer not to answer

C3. Did you have any drugs or personal belongings confiscated during your overdose? Select all that apply.

☐ No
☐ Yes, drugs confiscated
☐ Yes, personal belongings confiscated
☐ Don't Know
☐ Prefer not to answer

C4. Did anyone else on the scene have any drugs or personal belongings confiscated during your overdose? Select all that apply.

☐ No
☐ Yes, drugs confiscated
☐ Yes, personal belongings confiscated
☐ Don't Know
☐ Prefer not to answer

C5. Did you or any bystanders experience other legal consequences as a result of your most recent overdose?

◯ No
◯ Yes
◯ Don't Know
◯ Prefer not to answer

C6. What other legal consequences did you or any bystanders experience as a result of your most recent overdose?

____

Now we have some questions about services and/or resources you may have been offered after your most recent overdose.

S1. Refer to Page 11 of the Interview Booklet for the next question. After your most recent overdose, what services were you connected to? Please select all that apply.

- ☐ Emergency Department Transfer
- ☐ Crisis Solutions Center / Lane St near Dearborn & Rainier Street
- ☐ ORCA Center/Morrison on 3rd St
- ☐ Connections Kirkland
- ☐ Referred to an outpatient provider
- ☐ Connected to housing or shelter
- ☐ Connected to other social services
- ☐ Connected to another clinic
- ☐ Other
- ☐ No care connections made
- ☐ Don't Know
- ☐ Prefer not to answer

S2. Of the following options, who connected you to the Crisis Solutions Center?

- ☐ EMS Provider
- ☐ ED Provider
- ☐ Other
- ☐ Don't know
- ☐ Prefer not to answer

S2a. INTERVIEWER: Specify who connected the participant to the Crisis Solutions Center:

____

S2b. Approximately when did you first access the Crisis Solutions Center?

____

S2c S2c. How many times have you accessed the Crisis Solutions Center since your first visit?

____

Refer to page 12 of the interview booklet for the next question.

S2d. How satisfied were you with the Crisis Solutions Center?

- ○ Very satisfied
- ○ Satisfied
- ○ Neutral
- ○ Unsatisfied
- ○ Very unsatisfied
- ○ Prefer not to answer

S3. Of the following options, who connected you to the ORCA Center?

- ☐ EMS Provider
- ☐ ED Provider
- ☐ Other
- ☐ Don't know
- ☐ Prefer not to answer

S3a. INTERVIEWER: Specify who connected the participant to the ORCA Center:

____

S3b. Approximately when did you first access the ORCA Center?

____

S3c. How many times have you accessed the ORCA Center since your first visit?

____

Refer to page 12 of the interview booklet for the next question.

S3d. How satisfied were you with the ORCA Center?

- ◯ Very satisfied
- ◯ Satisfied
- ◯ Neutral
- ◯ Unsatisfied
- ◯ Very unsatisfied
- ◯ Prefer not to answer

S4. Of the following options, who connected you to Connections Kirkland?

- ☐ EMS Provider
- ☐ ED Provider
- ☐ Other
- ☐ Don't know
- ☐ Prefer not to answer

S4a. INTERVIEWER: Specify who connected the participant to Connections Kirkland:

____

S4b. Approximately when did you first access Connections Kirkland?

____

S4c. How many times have you accessed Connections Kirkland since your first visit?

____

Refer to page 12 of the interview booklet for the next question.

S4d. How satisfied were you with Connections Kirkland?

- ◯ Very satisfied
- ◯ Satisfied
- ◯ Neutral
- ◯ Unsatisfied
- ◯ Very unsatisfied
- ◯ Prefer not to answer

S5. Of the following options, who connected you to an outpatient provider?

- ☐ EMS Provider
- ☐ ED Provider
- ☐ Other
- ☐ Don't know
- ☐ Prefer not to answer

S5a. INTERVIEWER: Specify who connected the participant to an outpatient provider:

____

S5b. Approximately when did you first access the outpatient provider you were referred to?

____

S5c. How many times have you accessed the outpatient provider since your first visit?

____

Refer to page 12 of the interview booklet for the next question.

S5d. How satisfied were you with the outpatient provider?

- ◯ Very satisfied
- ◯ Satisfied
- ◯ Neutral
- ◯ Unsatisfied
- ◯ Very unsatisfied
- ◯ Prefer not to answer

S6. Of the following options, who connected you to housing or shelter?

- ☐ EMS Provider
- ☐ ED Provider
- ☐ Other
- ☐ Don't know
- ☐ Prefer not to answer

S6a. INTERVIEWER: Specify who connected the participant to housing or shelter:

____

S6b. Approximately when did you first access the housing or shelter you were referred to? ______________________

S6c. How many times have you accessed the housing or shelter service since your first visit? ______________________

Refer to page 12 of the interview booklet for the next question.

S6d. How satisfied were you with the housing or shelter service?

- ◯ Very satisfied
- ◯ Satisfied
- ◯ Neutral
- ◯ Unsatisfied
- ◯ Very unsatisfied
- ◯ Prefer not to answer

S7. Of the following options, who connected you to other social services?

- ☐ EMS Provider
- ☐ ED Provider
- ☐ Other
- ☐ Don't know
- ☐ Prefer not to answer

S7a. INTERVIEWER: Specify who connected the participant to other social services: ______________________

S7b. Approximately when did you first access to the social services you were referred to? ______________________

S7c. How many times have you accessed these social services since your first visit? ______________________

Refer to page 12 of the interview booklet for the next question.

S7d. How satisfied were you with the social services?

- ◯ Very satisfied
- ◯ Satisfied
- ◯ Neutral
- ◯ Unsatisfied
- ◯ Very unsatisfied
- ◯ Prefer not to answer

S8. Of the following options, who connected you to another clinic?

- ☐ EMS Provider
- ☐ ED Provider
- ☐ Other
- ☐ Don't know
- ☐ Prefer not to answer

S8a. INTERVIEWER: Specify who connected the participant to another clinic: ______________________

S8b. Approximately when did you first access the clinic you were referred to? ______________________

S8c. How many times have you accessed the clinic since your first visit? ______________________

Refer to page 12 of the interview booklet for the next question.

S8d. How satisfied were you with the clinic?

- ◯ Very satisfied
- ◯ Satisfied
- ◯ Neutral
- ◯ Unsatisfied
- ◯ Very unsatisfied
- ◯ Prefer not to answer

S9. What other services were you connected to? ______________________

S9a. Of the following options, who connected you to this service?	☐ EMS Provider<br>☐ ED Provider<br>☐ Other<br>☐ Don't know<br>☐ Prefer not to answer
S9b. INTERVIEWER: Specify who connected the participant to this service:	__________________________________
S9c. Approximately when did you first access the service you were referred to?	__________________________________
S9d. How many times have you accessed this service since your first visit?	__________________________________
Refer to page 12 of the interview booklet for the next question.<br><br>S9e. How satisfied were you with this service?	◯ Very satisfied<br>◯ Satisfied<br>◯ Neutral<br>◯ Unsatisfied<br>◯ Very unsatisfied<br>◯ Prefer not to answer
R1. Did you receive any of the following resources right after your overdose to take home with you? Please select all that apply. (INTERVIEWER: READ response options)	☐ Take-home Naloxone/Narcan<br>☐ Buprenorphine, also known as Suboxone or Subutex<br>☐ Fentanyl test strips<br>☐ Other<br>☐ None<br>☐ Unsure<br>☐ Prefer not to answer
R1a. What other resource did you receive right after your overdose?	__________________________________
R2. Refer to Page 13 of the Interview Booklet for the next question. As a result of your overdose, were you connected to any of the following medication-assisted treatments, or MAT?	☐ Buprenorphine, also known as Suboxone or Subutex<br>☐ Methadone<br>☐ Sublocade<br>☐ Naltrexone, also known as Vivitrol<br>☐ None<br>☐ Other<br>☐ Unsure<br>☐ Prefer not to answer
R2a. What other medication-assisted treatment (MAT) were you connected to?	__________________________________
Refer to page 14 of the interview booklet for the next question.<br><br>R3. On a scale from 1 to 5, with 1 being not at all valuable and 5 being very valuable, how valuable did you find the naloxone/narcan?	◯ Very valuable<br>◯ Valuable<br>◯ Neutral<br>◯ Unvaluable<br>◯ Very unvaluable<br>◯ Prefer not to answer
Refer to page 14 of the interview booklet for the next question.<br><br>R3a. On a scale from 1 to 5, with 1 being not at all valuable and 5 being very valuable, how valuable did you find the buprenorphine, also known as Suboxone or Subutex?	◯ Very valuable<br>◯ Valuable<br>◯ Neutral<br>◯ Unvaluable<br>◯ Very unvaluable<br>◯ Prefer not to answer

Refer to page 14 of the interview booklet for the next question.<br><br>R3b. On a scale from 1 to 5, with 1 being not at all valuable and 5 being very valuable, how valuable did you find the fentanyl test strips?	◯ Very valuable<br>◯ Valuable<br>◯ Neutral<br>◯ Unvaluable<br>◯ Very unvaluable<br>◯ Prefer not to answer
Refer to page 14 of the interview booklet for the next question.<br><br>R3c. On a scale from 1 to 5, with 1 being not at all valuable and 5 being very valuable, how valuable did you find the methadone?	◯ Very valuable<br>◯ Valuable<br>◯ Neutral<br>◯ Unvaluable<br>◯ Very unvaluable<br>◯ Prefer not to answer
Refer to page 14 of the interview booklet for the next question.<br><br>R3d. On a scale from 1 to 5, with 1 being not at all valuable and 5 being very valuable, how valuable did you find the sublocade?	◯ Very valuable<br>◯ Valuable<br>◯ Neutral<br>◯ Unvaluable<br>◯ Very unvaluable<br>◯ Prefer not to answer
Refer to page 14 of the interview booklet for the next question.<br><br>R3e. On a scale from 1 to 5, with 1 being not at all valuable and 5 being very valuable, how valuable did you find the naltrexone, also known as Vivitrol?	◯ Very valuable<br>◯ Valuable<br>◯ Neutral<br>◯ Unvaluable<br>◯ Very unvaluable<br>◯ Prefer not to answer
Refer to page 14 of the interview booklet for the next question.<br><br>R3f. On a scale from 1 to 5, with 1 being not at all valuable and 5 being very valuable, how valuable did you find the other resource you mentioned?	◯ Very valuable<br>◯ Valuable<br>◯ Neutral<br>◯ Unvaluable<br>◯ Very unvaluable<br>◯ Prefer not to answer
Refer to page 14 of the interview booklet for the next question.<br><br>R3g. On a scale from 1 to 5, with 1 being not at all valuable and 5 being very valuable, how valuable did you find the other MAT you mentioned?	◯ Very valuable<br>◯ Valuable<br>◯ Neutral<br>◯ Unvaluable<br>◯ Very unvaluable<br>◯ Prefer not to answer

Progress check: We've made it through about 50% of the interview questions.

Now we have some more questions about yourself. These questions are important because they help us to understand how similar the group of people in our study are to the wider population. Keep in mind that all your answers will be kept confidential.

D1. What is your current marital status?	◯ Single/Never married<br>◯ Married<br>◯ Separated<br>◯ Divorced<br>◯ Widow(er)<br>◯ Don't Know<br>◯ Prefer not to Answer

D2. What was the highest grade or degree you have completed in school?

- ◯ 8th grade or less (0-8)
- ◯ Some high school (9-11)
- ◯ High school graduate or GED (12)
- ◯ Some college or technical school (13-15)
- ◯ College graduate (16)
- ◯ Postgraduate Training or Degree (17+)
- ◯ Other (e.g. homeschooling)
- ◯ Unsure
- ◯ Prefer not to Answer

D3. Which of the following best describes your sexual orientation? You can select more than one.

- ☐ Heterosexual/straight
- ☐ Gay/lesbian
- ☐ Bisexual
- ☐ Pansexual
- ☐ Asexual
- ☐ Queer
- ☐ Another orientation not listed
- ☐ Unsure
- ☐ Prefer not to answer

D3a. How would you describe your sexual orientation?

____

For the next question, I am going to ask about your income. Keep in mind that factors such as income have a major impact on people's health and well-being and asking this question helps us determine the context of an individual's environment and its effect on health outcomes.

D4. Refer to Page 15 of the Interview Booklet for the next question. What are your current sources of income? Please remember that all answers will be kept confidential.

- ☐ Full-time employment
- ☐ Part-time employment
- ☐ Temporary work, including odd jobs, off-books, gigs, etc.
- ☐ Self-employment
- ☐ Unemployment benefits
- ☐ Other public assistance such as welfare, disability, or food stamps
- ☐ Spouse, partner, friend, or relative's income (including child support)
- ☐ Informal or street-based income
- ☐ Other
- ☐ No income
- ☐ Not sure
- ☐ Prefer not to answer

D4a. What is your other current source of income?

____

D5. What is your estimated income per week for full-time employment?

____

D5a. What is your estimated monthly income for full-time employment?

____

D6. What is your estimated income per week for part-time employment?

____

D6a. What is your estimated monthly income for part-time employment?

____

D7. What is your estimated income per week for temporary work, including odd jobs, off-books, gigs, etc.? ________________________________

D7a. What is your estimated monthly income for temporary work, including odd jobs, off-books, gigs, etc.? ________________________________

D8. What is your estimated income per week for self-employment? ________________________________

D8a. What is your estimated monthly income for self-employment? ________________________________

D9. What is your estimated income per week for unemployment benefits? ________________________________

D9a. What is your estimated monthly income for unemployment benefits? ________________________________

D10. What is your estimated income per week for other public assistance such as welfare, disability, or food stamps? ________________________________

D10a. What is your estimated monthly income for other public assistance such as welfare, disability, or food stamps? ________________________________

D11. What is your estimated income per week for spouse, partner, friend, or relative's income (including child support)? ________________________________

D11a. What is your estimated monthly income for spouse, partner, friend, or relative's income (including child support)? ________________________________

D12. What is your estimated income per week for informal or street-based income? ________________________________

D12a. What is your estimated monthly income for informal or street-based income? ________________________________

D13. What is your estimated income per week for your other current source of income? ________________________________

D13a. What is your estimated monthly income for informal or street-based income? ________________________________

D14. Refer to Page 16 of the Interview Booklet for the next 2 questions. Please indicate the number of the category that gives the best estimate of your personal annual income before taxes in the last year. This number should include the total amount from all of your income combined.

- ◯ [0] no personal income
- ◯ [1] under $5000
- ◯ [2] $5,000-$9,999
- ◯ [3] $10,000-$14,999
- ◯ [4] $15,000-$19,999
- ◯ [5] $20,000-$24,999
- ◯ [6] $25,000-$34,999
- ◯ [7] $35,000-$44,999
- ◯ [8] $45,000-$54,999
- ◯ [9] $55,000-$64,999
- ◯ [10] $65,000-$74,999
- ◯ [11] $75,000-$84,999
- ◯ [12] $85,000-$94,999
- ◯ [13] $95,000-$114,999
- ◯ [14] $115,000-$134,999
- ◯ [15] $135,000 and above
- ◯ [16] Don't know
- ◯ [17] Refused

D15. Using the same categories, please tell me the number that gives the best estimate of your total annual household income before taxes. By total household income I mean the total income for all of the people living in your home plus all other sources of income. Other sources of income would include such things as money market funds, social security, pensions, real estate, government entitlements, or alimony.

- ◯ [0] no income
- ◯ [1] under $5000
- ◯ [2] $5,000-$9,999
- ◯ [3] $10,000-$14,999
- ◯ [4] $15,000-$19,999
- ◯ [5] $20,000-$24,999
- ◯ [6] $25,000-$34,999
- ◯ [7] $35,000-$44,999
- ◯ [8] $45,000-$54,999
- ◯ [9] $55,000-$64,999
- ◯ [10] $65,000-$74,999
- ◯ [11] $75,000-$84,999
- ◯ [12] $85,000-$94,999
- ◯ [13] $95,000-$114,999
- ◯ [14] $115,000-$134,999
- ◯ [15] $135,000 and above
- ◯ [16] Don't know
- ◯ [17] Refused

The next questions are about health insurance. By health insurance, we mean health plans people get through employment or purchased directly, as well as government programs like Medicare and Medicaid that provide medical care or help pay medical bills.

D16. Do you have health insurance?

- ◯ No
- ◯ Yes
- ◯ Not sure
- ◯ Prefer not to answer

D17. Refer to Page 17 of the Interview Booklet for the next question. What kind of health insurance do you have?

- ☐ Medicare, a public health insurance program for people 65 and older and disabled persons
- ☐ Medicaid, a public health insurance program for people with low incomes (including Apple Health)
- ☐ Any military health care plans such as Tri-Care, CHAMPUS or CHAMP-VA
- ☐ Any private health care plans like an HMO (not including a Medicare HMO), Blue Cross/Blue Shield, or other company
- ☐ Some other health insurance
- ☐ Don't Know
- ☐ Prefer not to answer

D17a. What other health insurance do you have?

____

The next set of questions are about your housing or where you sleep most often.

D18. In the last 6 months, have you had to live somewhere that you did not want to live?	◯ No<br>◯ Yes<br>◯ Unsure<br>◯ Prefer not to answer
D20. In the last 6 months, have you been unhoused or had to live with family or friends to avoid being unhoused?	◯ No<br>◯ Yes<br>◯ Unsure<br>◯ Prefer not to answer
D19. Do you expect to stay in your current place for the next 6 months?	◯ No<br>◯ Yes<br>◯ Unsure<br>◯ Prefer not to answer
D21. In the last 6 months, have you had difficulty paying (or were you unable to pay) for housing?	◯ No<br>◯ Yes<br>◯ Unsure<br>◯ Prefer not to answer
D22. In the last 6 months have you had trouble getting housing?	◯ No<br>◯ Yes<br>◯ Unsure<br>◯ Prefer not to answer
How many times have you moved in the last 6 months? Please include any times you may have been involuntarily moved due to encampment site removal, RV site removal, and/or eviction.	__________________________
D23. Did your most recent overdose occur within 30 days of an encampment site removal, RV site removal, or eviction?	◯ No<br>◯ Yes<br>◯ Unsure<br>◯ Prefer not to answer
D24. Which of the following has impacted you in the past 6 months? Select all that apply.	☐ Encampment removal<br>☐ RV site removal<br>☐ Eviction<br>☐ Other<br>☐ Don't know<br>☐ Prefer not to answer
D24a. In what other way have you been involuntarily moved?	__________________________
D25. How many times have you been moved involuntarily (e.g. encampment site removal, RV site removal, eviction) in the last 6 months?	__________________________
D26. Refer to Page 18 of the Interview Booklet for the next question. How likely is it, do you think, that you will be able to pay for your housing (e.g. rent/mortgage) this month?	◯ Very unlikely<br>◯ Unlikely<br>◯ Neither likely nor unlikely<br>◯ Likely<br>◯ Very likely<br>◯ Do not pay for housing<br>◯ Unsure<br>◯ Prefer not to answer

D27. Refer to Page 19 of the Interview Booklet for the next question. When you think of your financial situation overall, how often are the following needs adequately met? Refer to flashcard for this question. You can respond with "Never," "Some of the time," or "Always."

	Never	Some of the time	Always	Unsure	Prefer not to answer
Food for two meals a day	◯	◯	◯	◯	◯
House or apartment	◯	◯	◯	◯	◯
Money to buy necessities	◯	◯	◯	◯	◯
Enough clothes for you or your family	◯	◯	◯	◯	◯
Heat for your house or apartment	◯	◯	◯	◯	◯
Indoor plumbing or water	◯	◯	◯	◯	◯
Money to pay monthly bills	◯	◯	◯	◯	◯
Good job for yourself or your spouse	◯	◯	◯	◯	◯
Medical care for you or your family	◯	◯	◯	◯	◯
Public assistance (SSI, AFDC, Medicaid, etc.)	◯	◯	◯	◯	◯
Dependable transportation	◯	◯	◯	◯	◯
Time to get enough sleep/rest	◯	◯	◯	◯	◯
Furniture for your home or apartment	◯	◯	◯	◯	◯
Telephone or access to phone	◯	◯	◯	◯	◯
Dental care for you or your family	◯	◯	◯	◯	◯
Money to buy things for yourself	◯	◯	◯	◯	◯
Money for entertainment	◯	◯	◯	◯	◯
Money to save	◯	◯	◯	◯	◯

The next set of questions will again ask you about experiences you may have had with the criminal justice system.

C7. Not counting minor traffic violations, have you ever been arrested or booked for breaking the law? [Note: Being 'booked' means that you were taken into custody and processed by the police or by someone with the courts, even if you were then released.]

◯ No
◯ Yes
◯ Unsure
◯ Prefer not to answer

C9. Have you ever spent time in a city or county jail?

◯ No
◯ Yes
◯ Unsure
◯ Prefer not to answer

C8. Have you ever spent time in a federal or state prison?

◯ No
◯ Yes
◯ Unsure
◯ Prefer not to answer

READ: The next questions ask you to specify the types of offenses you were arrested and booked for within the past 6 months.

C10. Not counting minor traffic violations, how many times during the past 6 months have you ever been arrested or booked for breaking the law?	______________________
C10a. Can you please indicate the type(s) of charges?	☐ Driving under the influence of alcohol or drugs or for drunkenness or other liquor law regulations<br>☐ Possession, manufacture, or sale of drugs<br>☐ Prostitution or commercialized sex<br>☐ Fraud, possessing stolen goods, arson, or vandalism<br>☐ Any other charge (including aggravated assault, simple assault, battery, or forcible rape, murder, homicide, or nonnegligent manslaughter, motor vehicle theft or larceny, burglary, breaking and entering, or robbery)<br>☐ Unsure<br>☐ Prefer not to answer
C11. In the past 6 months, were you arrested and booked for driving under the influence of alcohol or drugs or for drunkenness or other liquor law violations?	◯ No<br>◯ Yes<br>◯ Unsure<br>◯ Prefer not to answer
C12. In the past 6 months, were you arrested and booked for possession, manufacture, or sale of drugs?	◯ No<br>◯ Yes<br>◯ Unsure<br>◯ Prefer not to answer
C13. In the past 6 months, were you arrested and booked for prostitution or commercialized sex?	◯ No<br>◯ Yes<br>◯ Unsure<br>◯ Prefer not to answer
C14. In the past 6 months, were you arrested and booked for fraud, possessing stolen goods, arson, or vandalism?	◯ No<br>◯ Yes<br>◯ Unsure<br>◯ Prefer not to answer
C15. In the past 6 months, were you arrested and booked for any other charge (including aggravated assault, simple assault, battery, or forcible rape, murder, homicide, or nonnegligent manslaughter, motor vehicle theft or larceny, burglary, breaking and entering, or robbery)	◯ No<br>◯ Yes<br>◯ Unsure<br>◯ Prefer not to answer

Now we have some questions about drugs you may have used in the past 2 months. Again, please keep in mind that all your answers are confidential. Refer to flashcard for the next 2 questions.

SS1. Refer to Page 20 of the Interview Booklet for the next questions. Which of the following drugs or substances have you used in the past 2 months? Please select all that apply.	☐ Methamphetamine<br>☐ Cocaine or crack<br>☐ Benzos/downers like Valium or Xanax<br>☐ Heroin<br>☐ Fentanyl<br>☐ Cannabis<br>☐ Alcohol<br>☐ Drugs you mix and use together (like speedball or goofball)<br>☐ Another drug not listed<br>☐ None of the above<br>☐ Unsure<br>☐ Prefer not to answer

SS2. Which of the following drugs or substances have you used in the past 7 days? Please select all that apply.	☐ Methamphetamine<br>☐ Cocaine or crack<br>☐ Benzos/downers like Valium or Xanax<br>☐ Heroin<br>☐ Fentanyl<br>☐ Cannabis<br>☐ Alcohol<br>☐ Drugs you mix and use together (like speedball or goofball)<br>☐ Another drug not listed<br>☐ None of the above<br>☐ Unsure<br>☐ Prefer not to answer
SS2a. Please specify which drugs you mixed and used together in the past 2 months.	__________________________________
SS2b. Please specify which other drug or drugs you used in the past 2 months.	__________________________________
SS3. In the past week, how many days did you use methamphetamine?	◯ 1 day<br>◯ 2 days<br>◯ 3 days<br>◯ 4 days<br>◯ 5 days<br>◯ 6 days<br>◯ 7 days<br>◯ Unsure<br>◯ Prefer not to answer
SS3a. In the past week, how did you use methamphetamine? Please select all that apply.	☐ Snort<br>☐ Inject<br>☐ Smoke<br>☐ Ingest<br>☐ Boof<br>☐ Unsure<br>☐ Prefer not to answer
SS4. In the past week, how many days did you use cocaine or crack?	◯ 1 day<br>◯ 2 days<br>◯ 3 days<br>◯ 4 days<br>◯ 5 days<br>◯ 6 days<br>◯ 7 days<br>◯ Unsure<br>◯ Prefer not to answer
SS4a. In the past week, how did you use cocaine or crack? Please select all that apply.	☐ Snort<br>☐ Inject<br>☐ Smoke<br>☐ Ingest<br>☐ Boof<br>☐ Unsure<br>☐ Prefer not to answer

SS5. In the past week, how many days did you use benzos/downers like Valium or Xanax?	◯ 1 day<br>◯ 2 days<br>◯ 3 days<br>◯ 4 days<br>◯ 5 days<br>◯ 6 days<br>◯ 7 days<br>◯ Unsure<br>◯ Prefer not to answer
SS5a. In the past week, how did you use benzos/downers like Valium or Xanax? Please select all that apply.	☐ Snort<br>☐ Inject<br>☐ Smoke<br>☐ Ingest<br>☐ Boof<br>☐ Unsure<br>☐ Prefer not to answer
SS6. In the past week, how many days did you use heroin?	◯ 1 day<br>◯ 2 days<br>◯ 3 days<br>◯ 4 days<br>◯ 5 days<br>◯ 6 days<br>◯ 7 days<br>◯ Unsure<br>◯ Prefer not to answer
SS6a. In the past week, how did you use heroin? Please select all that apply.	☐ Snort<br>☐ Inject<br>☐ Smoke<br>☐ Ingest<br>☐ Boof<br>☐ Unsure<br>☐ Prefer not to answer
SS7. In the past week, how many days did you use fentanyl?	◯ 1 day<br>◯ 2 days<br>◯ 3 days<br>◯ 4 days<br>◯ 5 days<br>◯ 6 days<br>◯ 7 days<br>◯ Unsure<br>◯ Prefer not to answer
SS7a. In the past week, how did you use fentanyl? Please select all that apply.	☐ Snort<br>☐ Inject<br>☐ Smoke<br>☐ Ingest<br>☐ Boof<br>☐ Unsure<br>☐ Prefer not to answer

SS8. In the past week, how many days did you use cannabis?	◯ 1 day<br>◯ 2 days<br>◯ 3 days<br>◯ 4 days<br>◯ 5 days<br>◯ 6 days<br>◯ 7 days<br>◯ Unsure<br>◯ Prefer not to answer
SS8a. In the past week, how did you use cannabis? Please select all that apply.	☐ Smoke<br>☐ Ingest<br>☐ Unsure<br>☐ Prefer not to answer
SS9. In the past week, how many days did you drink alcohol?	◯ 1 day<br>◯ 2 days<br>◯ 3 days<br>◯ 4 days<br>◯ 5 days<br>◯ 6 days<br>◯ 7 days<br>◯ Unsure<br>◯ Prefer not to answer
SS10. In the past week, how many days did you mix or use drugs together?	◯ 1 day<br>◯ 2 days<br>◯ 3 days<br>◯ 4 days<br>◯ 5 days<br>◯ 6 days<br>◯ 7 days<br>◯ Unsure<br>◯ Prefer not to answer
SS10a. In the past week, how did you mix or use drugs together? Please select all that apply.	☐ Snort<br>☐ Inject<br>☐ Smoke<br>☐ Ingest<br>☐ Boof<br>☐ Unsure<br>☐ Prefer not to answer
SS11. How old were you when you first used one of the drugs we just asked about?	________________________
SS12. How old were you when you first had an alcoholic beverage?<br><br>If participant notes that they have never drunk alcohol, write "NA."	________________________

SS13. Which one of these is your main substance or the substance that you use most often? Please only select one and refer to flashcard for this question.	◯ Methamphetamine<br>◯ Cocaine or crack<br>◯ Benzos/downers like Valium or Xanax<br>◯ Heroin<br>◯ Fentanyl<br>◯ Cannabis<br>◯ Alcohol<br>◯ Drugs you mix and use together (like speedball or goofball)<br>◯ Another drug not listed<br>◯ Unsure<br>◯ Prefer not to answer
SS13a. What other substance is your main substance or the substance you use most often?	__________________________

Progress check: We've finished about 75% of the interview questions.

READ: Next, I'm going to ask you about your experiences in receiving treatment for substance misuse. By treatment, I mean you participated in a program or took medicine to treat your drug use. This includes a variety of things, including outpatient, inpatient, residential, detox, 12-step program, and more, and can include treatment for alcohol use."

T1. Have you ever received help or treatment for substance misuse?	◯ No<br>◯ Yes<br>◯ Unsure<br>◯ Prefer not to answer
T2. At what age did you first receive help or treatment for substance misuse?	__________________________
T3. Have you ever received outpatient treatment (e.g. group or individual therapy, appointments with a medical provider or clinic)?	◯ No<br>◯ Yes<br>◯ Unsure<br>◯ Prefer not to answer
T3a. Are you currently receiving outpatient treatment (e.g., group or individual therapy, appointments with a medical provider or clinic)?	◯ No<br>◯ Yes<br>◯ Unsure<br>◯ Prefer not to answer
T4. Have you ever received inpatient treatment (e.g. inpatient detox, residential program, hospital admission)?	◯ No<br>◯ Yes<br>◯ Unsure<br>◯ Prefer not to answer
T4a. Are you currently receiving inpatient treatment (e.g., inpatient detox, residential program, hospital admission)?	◯ No<br>◯ Yes<br>◯ Unsure<br>◯ Prefer not to answer
T5. Have you ever received medication to treat substance misuse?	◯ No<br>◯ Yes<br>◯ Unsure<br>◯ Prefer not to answer
T5a. Are you currently taking any medications to treat substance misuse?	◯ No<br>◯ Yes<br>◯ Unsure<br>◯ Prefer not to answer

T5b. Which of the following medications were you prescribed? Select all that apply. Response options are on Page 12 of the Interview Booklet.	☐ Methadone<br>☐ Buprenorphine, also known as Suboxone or Subutex<br>☐ Naltrexone, also known as Vivitrol<br>☐ Sublocade<br>☐ Other<br>☐ Unsure<br>☐ Prefer not to answer
T5c. What other medication were you prescribed?	__________________________________
T6. Regarding substance misuse and/or treatment, did you seek help from any of the following people? Select all that apply.	☐ A general practitioner or family doctor<br>☐ A psychiatrist, psychologist, or other mental health professional<br>☐ Another health professional<br>☐ A minister, priest, or rabbi<br>☐ Friends<br>☐ Family<br>☐ Someone else<br>☐ None of the above
T7. Refer to Page 21 of the Interview Booklet for the next question. Did you seek help at any of the following places?	☐ A hospital emergency room<br>☐ A psychiatric outpatient clinic<br>☐ A drug or alcohol clinic<br>☐ Crisis center or hotline<br>☐ Syringe service program<br>☐ A self-help group where people with similar difficulties help one another<br>☐ A mental health clinic<br>☐ Some other organization<br>☐ None of the above
T8. Have you ever received any other treatment we haven't already discussed?	○ No<br>○ Yes
T8a. What other treatment have you received?	__________________________________

The next set of questions are about your mental health.

MH1. For this next set of questions, refer to Page 22 of the Interview Booklet. Please indicate over the last 2 weeks how often you have been bothered by any of the following problems. You can answer not at all, several days, more than half the days, or nearly every day.

MH1a. Little interest or pleasure in doing things

- ◯ Not at all
- ◯ Several days
- ◯ More than half the days
- ◯ Nearly every day
- ◯ Unsure
- ◯ Prefer not to answer

MH1b. Feeling down, depressed, or hopeless

- ◯ Not at all
- ◯ Several days
- ◯ More than half the days
- ◯ Nearly every day
- ◯ Unsure
- ◯ Prefer not to answer

MH1c. Feeling nervous, anxious, or on edge

- ◯ Not at all
- ◯ Several days
- ◯ More than half the days
- ◯ Nearly every day
- ◯ Unsure
- ◯ Prefer not to answer

MH1d. Not being able to stop or control worrying

- ◯ Not at all
- ◯ Several days
- ◯ More than half the days
- ◯ Nearly every day
- ◯ Unsure
- ◯ Prefer not to answer

Trouble falling or staying asleep, or sleeping too much

- ◯ Not at all
- ◯ Several days
- ◯ More than half the days
- ◯ Nearly every day
- ◯ Unsure
- ◯ Prefer not to answer

Feeling tired or having little energy

- ◯ Not at all
- ◯ Several days
- ◯ More than half the days
- ◯ Nearly every day
- ◯ Unsure
- ◯ Prefer not to answer

Poor appetite or overeating

- ◯ Not at all
- ◯ Several days
- ◯ More than half the days
- ◯ Nearly every day
- ◯ Unsure
- ◯ Prefer not to answer

Feeling bad about yourself - or that you are a failure or have let yourself or your family down.

- ◯ Not at all
- ◯ Several days
- ◯ More than half the days
- ◯ Nearly every day
- ◯ Unsure
- ◯ Prefer not to answer

Trouble concentrating on things such as reading the newspaper or watching television	◯ Not at all<br>◯ Several days<br>◯ More than half the days<br>◯ Nearly every day<br>◯ Unsure<br>◯ Prefer not to answer
Moving or speaking so slowly that other people could have noticed? Or the opposite - being so fidgety or restless that you have been moving around a lot more than usual	◯ Not at all<br>◯ Several days<br>◯ More than half the days<br>◯ Nearly every day<br>◯ Unsure<br>◯ Prefer not to answer
Thoughts that you would be better off dead or hurting yourself in some way	◯ Not at all<br>◯ Several days<br>◯ More than half the days<br>◯ Nearly every day<br>◯ Unsure<br>◯ Prefer not to answer
MH2. Have you ever received a mental health diagnosis?	◯ No<br>◯ Yes<br>◯ Unsure<br>◯ Prefer not to answer
MH2a. I'm going to read a list of mental health diagnoses. Which ones, if any, have you been diagnosed with? Select all that apply.	☐ Depression<br>☐ Anxiety<br>☐ Bipolar disorder<br>☐ Schizophrenia<br>☐ Personality disorder<br>☐ Something else<br>☐ Unsure<br>☐ Prefer not to answer
MH2b. Please describe which other mental health diagnosis you have received.	________________________
MH3. How old were you when you were diagnosed with depression?	________________________
MH3a. Are you currently or have you ever received outpatient treatment for depression?	◯ Yes, currently<br>◯ Yes, previously<br>◯ No<br>◯ Unsure<br>◯ Prefer not to answer
MH3b. Are you currently or have you ever received inpatient treatment for depression?	◯ Yes, currently<br>◯ Yes, previously<br>◯ No<br>◯ Unsure<br>◯ Prefer not to answer
MH3c. Are you currently or have you ever received medication for depression?	◯ Yes, currently<br>◯ Yes, previously<br>◯ No<br>◯ Unsure<br>◯ Prefer not to answer

MH3d. What medications were you prescribed for depression? __________________________________

MH3e. Have you received any other treatment for depression? __________________________________

MH4. How old were you when you were diagnosed with anxiety? __________________________________

MH4a. Are you currently or have you ever received outpatient treatment for anxiety?
- ◯ Yes, currently
- ◯ Yes, previously
- ◯ No
- ◯ Unsure
- ◯ Prefer not to answer

MH4b. Are you currently or have you ever received inpatient treatment for anxiety?
- ◯ Yes, currently
- ◯ Yes, previously
- ◯ No
- ◯ Unsure
- ◯ Prefer not to answer

MH4c. Are you currently or have you ever received medication for anxiety?
- ◯ Yes, currently
- ◯ Yes, previously
- ◯ No
- ◯ Unsure
- ◯ Prefer not to answer

MH4d. What medications were you prescribed for anxiety? __________________________________

MH4e. Have you received any other treatment for anxiety? __________________________________

MH5. How old were you when you were diagnosed with bipolar disorder? __________________________________

MH5a. Are you currently or have you ever received outpatient treatment for bipolar disorder?
- ◯ Yes, currently
- ◯ Yes, previously
- ◯ No
- ◯ Unsure
- ◯ Prefer not to answer

MH5b. Are you currently or have you ever received inpatient treatment for bipolar disorder?
- ◯ Yes, currently
- ◯ Yes, previously
- ◯ No
- ◯ Unsure
- ◯ Prefer not to answer

MH5c. Are you currently or have you ever received medication for bipolar disorder?
- ◯ Yes, currently
- ◯ Yes, previously
- ◯ No
- ◯ Unsure
- ◯ Prefer not to answer

MH5d. What medications were you prescribed for bipolar disorder? __________________________________

MH5e. Have you received any other treatment for bipolar disorder? __________________________________

MH6. How old were you when you were diagnosed with schizophrenia?	__________________________
MH6a. Are you currently or have you ever received outpatient treatment for schizophrenia?	◯ Yes, currently<br>◯ Yes, previously<br>◯ No<br>◯ Unsure<br>◯ Prefer not to answer
MH6b. Are you currently or have you ever received inpatient treatment for schizophrenia?	◯ Yes, currently<br>◯ Yes, previously<br>◯ No<br>◯ Unsure<br>◯ Prefer not to answer
MH6c. Are you currently or have you ever received medication for schizophrenia?	◯ Yes, currently<br>◯ Yes, previously<br>◯ No<br>◯ Unsure<br>◯ Prefer not to answer
MH6d. What medications were you prescribed for schizophrenia?	__________________________
MH6e. Have you received any other treatment for schizophrenia?	__________________________
MH7. How old were you when you were diagnosed with a personality disorder?	__________________________
MH7a. Are you currently or have you ever received outpatient treatment for a personality disorder?	◯ Yes, currently<br>◯ Yes, previously<br>◯ No<br>◯ Unsure<br>◯ Prefer not to answer
MH7b. Are you currently or have you ever received inpatient treatment for a personality disorder?	◯ Yes, currently<br>◯ Yes, previously<br>◯ No<br>◯ Unsure<br>◯ Prefer not to answer
MH7c. Are you currently or have you ever received medication for a personality disorder?	◯ Yes, currently<br>◯ Yes, previously<br>◯ No<br>◯ Unsure<br>◯ Prefer not to answer
MH7d. What medications were you prescribed for a personality disorder?	__________________________
MH7e. Have you received any other treatment for a personality disorder?	__________________________
MH8. How old were you when you were diagnosed with the other mental health diagnosis you described?	__________________________

MH8a. Are you currently or have you ever received outpatient treatment for the other mental health diagnosis you described?

- ◯ Yes, currently
- ◯ Yes, previously
- ◯ No
- ◯ Unsure
- ◯ Prefer not to answer

MH8b. Are you currently or have you ever received inpatient treatment for the other mental health diagnosis you described?

- ◯ Yes, currently
- ◯ Yes, previously
- ◯ No
- ◯ Unsure
- ◯ Prefer not to answer

MH8c. Are you currently or have you ever received medication for the other mental health diagnosis you described?

- ◯ Yes, currently
- ◯ Yes, previously
- ◯ No
- ◯ Unsure
- ◯ Prefer not to answer

MH8d. What medications were you prescribed for the other mental health diagnosis you described?

____

MH8e. Have you received any other treatment for the other mental health diagnosis you described?

____

MH9. In general, would you say your health is:

- ◯ Excellent
- ◯ Very Good
- ◯ Good
- ◯ Fair
- ◯ Poor
- ◯ Unsure
- ◯ Prefer not to answer

MH10. Thinking back to a year ago, would you say that your health since then has:

- ◯ Considerably improved
- ◯ Somewhat improved
- ◯ Stayed about the same
- ◯ Somewhat worsened
- ◯ Considerably worsened
- ◯ Unsure
- ◯ Prefer not to answer

Progress check: We are almost done with our questions.

Check-in: We've answered some tough questions. I want to check in and see how you are doing. As a reminder, we will have time after the interview to debrief if you would like.

We just have a few more about EMS and your past overdose experiences.

O4. How many times in your life have you had an overdose?

____

O4a. How many of those times was EMS called to respond to your overdose?

____

O4b. Of the times that EMS was called, how many times did EMS actually respond to your overdose?

____

O5. Refer to Page 23 of the Interview Booklet for the next question. If you were with someone who had an overdose, when would you call EMS to respond? Select all that apply.	☐ I would call EMS every time<br>☐ I would only call if someone else wasn't able to give Naloxone or Narcan<br>☐ I would only call if administering Naloxone or Narcan wasn't working to reverse the overdose<br>☐ I would only call if I did not feel worried about arrest (e.g., if I didn't have a warrant, if I didn't have drugs or a weapon with me)<br>☐ I would call and try to leave before EMS arrived<br>☐ I would never call EMS<br>☐ Unsure<br>☐ Prefer not to answer
O6. How many times in your life have you witnessed someone else having an overdose?	__________________________________
O6a. How many times in your life have you called EMS for someone else who had an overdose?	__________________________________
O6b. Of the times that EMS was called, how many times did EMS actually respond when you called about someone else's overdose?	__________________________________

We know that sometimes people didn't mean to overdose, or they overdose by accident. Other times, people overdose on purpose or intentionally, and then there are times where people might be indifferent or might not care whether they overdose while using drugs.

O7. Have you ever had an overdose that was on purpose or intentional?
- ◯ No
- ◯ Yes
- ◯ Unsure
- ◯ Prefer not to answer

O7a. Was your most recent overdose on purpose or intentional?
- ◯ No
- ◯ Yes
- ◯ Unsure
- ◯ Prefer not to answer

O8. Have you ever had an overdose where you were indifferent or did not care about the possibility of overdosing?
- ◯ No
- ◯ Yes
- ◯ Unsure
- ◯ Prefer not to answer

O8a. Was your most recent overdose a case where you were indifferent or did not care about the possibility of overdosing?
- ◯ No
- ◯ Yes
- ◯ Unsure
- ◯ Prefer not to answer

E8. What would you want an EMS provider to know about you as a person so that they could provide you with the best care possible?

____

Is there anything else you would like to tell me before we finish?

____

Thank you so much for taking the time to participate in our ORCID study survey. These responses will be used to help understand and improve emergency services and overdose responses. If you have any further questions, you can contact us using the information on the consent form.

HIDDEN, AUTOMATIC: End time of questionnaire

____

Before we finish, I want to let you know that we will be following up with some people to do a more in-depth interview, which is worth $100. In order to follow up with you, can you please provide me with your contact information, starting with your phone number?

____

What is your email address?

____

Lastly, in order to complete our evaluation of recent changes to EMS protocols, we are requesting additional to help us find out how these changes were implemented in the field. We will use a secondary data set from King County EMS to look at the impact of these changes on population-level outcomes. What is your first name?

____

Middle name?

____

Last name?

____

What is your home address?	__________________________________
What is your social security number?	__________________________________
END1. INTERVIEWER: Please confirm. Did the person complete the survey?	◯ Did NOT complete the survey<br>◯ DID complete the survey
END2. INTERVIEWER: Do you have any comments to add?	◯ No<br>◯ Yes
END3. Enter interviewer comments.	__________________________________
