# Supplementary material for: Overdose response centering inequity and diversity study: a protocol for assessing the population-level and equity impact of the emergency medical services system changes using critical race theory
Source: Front Public Health. 2025 Sep 15;13:1629518. doi: 10.3389/fpubh.2025.1629518 (PMC12477233; doi:10.3389/fpubh.2025.1629518)
Supplement: Supplementary file 3 [file Data_Sheet_3.PDF]

ORCID AIM 2 INTERVIEW GUIDE

Interviewer: [if you have not already introduced yourself] Hello. My name is [name] and I am with Research with Expert Advisors on Drug Use at the University of Washington.

Thank you for participating in this interview for the Overdose Response Centering Inequity and Diversity (ORCID) study. In this study, we are evaluating recent changes in overdose response in the Emergency Medical System to learn about their impact on overdose experiences of people of color, especially Black and Indigenous people. Our goal with this study is to learn how EMS is working for people who overdose and what should be changed about EMS overdose response, so it works best for all people who use drugs.

This interview will last about an hour. In your first survey with us, we asked you some short-answer questions about your experiences, including some information that was not directly related to your experiences with overdose, such as your housing status. In this interview, we will be focusing on a few of the same themes as we did during your survey, but this time we'd like you to answer with as much detail as you can. We will start by asking some questions to learn about you. Most of our questions will focus on your experiences with EMS, like firefighters, paramedics, and law enforcement after an overdose. We will ask you to share what you remember about what happened during those experiences, but also about your thoughts and feelings about them. We will also ask questions about your preferences during those encounters.

We understand that asking about your experiences, including experiences related to substance use, overdose, discrimination, and criminalization, may feel uncomfortable or upsetting. Please feel free to ask any questions you have during this interview or skip any questions that you do not wish to answer. If you need a break or if you wish to end the interview, you are free to do so at any time. If there is anything else you need to feel comfortable during this interview, please let us know. Remember that there are no wrong answers to any of these questions. We want to understand your perspective and opinions based on your experience.

[pause and ask if they have any questions].

As mentioned in the informed consent, this interview will be audio recorded. The recording will not be shared with anyone outside of the study team. Are you ok if I start the recording now?

[start recording] To confirm, I have started the recording.

Section 1. Overall experiences with EMS

First, we want to ask you some questions to get to know your overall experiences with EMS and overdose.

1. Can you tell me a little about yourself and your day-to-day life right now?

2. Can you share your thoughts or how you typically think about emergency medical services, or EMS, including paramedics, firefighters, and law enforcement?

3. What has a typical interaction with paramedics and firefighters been like for you?

4. What has a typical interaction with law enforcement been like for you?

**Section 2. Recent overdose experiences**

Thank you for answering those questions. Now we're going to ask you to tell us a bit more about your recent experiences with EMS after an overdose. We are interested in hearing about your most recent overdose, but if there are others in the last 6 months you'd like to share about, that's okay too.

2. Can you walk me through what happened the last time EMS responded to you after an overdose?
    - Was anyone else there besides EMS? What were they doing before/during/after your overdose?

3. What happened after EMS arrived - where did you go, and who made the decisions about that?

**Section 3. Health care experiences, including discrimination & medical racism**

Thanks for sharing those experiences with me. In the next section, I'm going to ask you some more questions about how you felt during your encounter with EMS after your most recent overdose.

4. Overall, can you describe how you felt during the interaction you had with EMS after your most recent overdose?
    - Did you feel safe? Respected? Were you able to ask questions or express concern?

5. Do you feel like you were treated differently because of who you are or how you were perceived?
    - What happened that made you feel that way?
    - Have you had different experiences in other situations with EMS?

6. Have you ever felt that your race, ethnicity, or language influenced the way you were treated during an EMS encounter?
    - Was that during a recent experience or others?

7. Were there any comments or actions that stood out to you, either positive or negative?
    - Do you feel that EMS providers made any assumptions about you during that interaction?
    - How did your experience affect your trust in responders?

8. Based on what you've experienced, do you think people from different backgrounds or neighborhoods are treated the same way by EMS?

9. What could EMS have done differently during that experience to make it better for you?

Section 4. Resources and services offered

Thanks for sharing your experiences with me. In the next couple of sections, I'm going to ask you some more specific questions about services you may have been offered after your most recent overdose. Examples of services include: detox, long-term treatment, peer support, post overdose/ORCA center, medications for opioid use disorder like buprenorphine or methadone, harm reduction services, etc.)

10. Can you tell me about any resources or services you were offered after your most recent overdose?
    - Who offered these resources/services?
    - How and when did they offer them?

11. What were the reasons you chose to accept or not accept the resources/services that were offered?
    - What influenced your decision the most?
    - Can you tell me how you felt about those services and resources at the time?
    - Was there anything about the way EMS presented it that influenced your decision?

12. What did you find valuable about those resources after using them? How did you use them?
    - How helpful did you feel they were in the long-term, and why?

13. What services would have been most helpful after your overdose?
    - What are the biggest barriers you face when trying to access services that you are interested in?
    - EMS teams have started offering buprenorphine (suboxone) to people after they overdose to reduce their withdrawal symptoms and prevent future overdoses. How interested would you have been in that service? Why?

Section 5. Experiences with law enforcement

Thank you for sharing your perspective. In this next set of questions, I'd like to learn about your experiences with law enforcement and immigration enforcement (ICE).

14. How have your experiences with law enforcement/ICE impacted your ability or desire to access other services related to your substance use?

15. What, if anything, could change about the laws or law enforcement that would have the biggest positive impact on people who use drugs right now?

- What kind of law or policy changes would make you feel safe calling 911 for help during an overdose?

Section 6. Experiences with encampment sweeps

Sometimes, things like policies, laws, or the way public spaces are managed can make it harder for people to stay safe or get what they need. These are called structural barriers. That could include things like not having enough shelters, lack of affordable housing, or how outdoor spaces are managed.

16. Have you experienced any structural barriers that made it harder for you to stay safe, access resources, or avoid overdose?
    - Are there specific examples that stand out?

Encampment removals, sometimes called sweeps, are when the city or other agencies clear out places where people are living outside. That can mean asking people to leave, throwing away belongings, or removing tents and shelters.

17. If you have experienced an encampment removal, how did that affect your ability to stay safe, access resources, or prevent overdose?

Section 7. Wrap Up

Thank you so much. We're almost done. This last set of questions is about advice that you might have for us.

18. During this study we did several surveys. What do you remember about those surveys?

19. What do you think would be a good way to share the findings from this study?

20. Is there anything else you want to share with us?

Thank you so much for your time. I'm turning off the recorder now.
